# Depression detectives: piloting a methodology for online co-produced research

**DOI:** 10.1186/s40900-026-00889-2

**Published:** 2026-05-18

**Authors:** Sophia Collins, Christine Kupfer, Stephanie Organ, Melissa Lewins, Andrew McIntosh, Iona Beange, Collaboration Group: Depression Detectives. Edinburgh Neuroscience

**Affiliations:** 1 Science is People Ltd, Lauder, Berwickshire, Scotland, UK; 2 University of Aberdeen, Aberdeen, Scotland, UK; 3 Freelance Science Communicator and Researcher, Cheltenham, UK; 4 Institute for Neuroscience and Cardiovascular Research, University of Edinburgh, Edinburgh, UK; 5 UK Biobank, Stockport, England, UK; 6 School of Population Health Sciences, University of Edinburgh, Edinburgh, UK

**Keywords:** Depression, Co-production, Co-creation, Citizen science, General practitioners, Data science, Big data, Patient involvement, Mental health, Patient and public involvement

## Abstract

**Background:**

Incorporating lived experiences into research is claimed to enhance depth, relevance, real-world applicability and equity. However, data scientists have been relatively slow to adopt this approach. The “Depression Detectives” (DD) project aimed to bridge this gap by connecting Big Data researchers with individuals with lived experience of depression. Depression is both a significant global health issue and a deeply personal experience. Feelings of isolation often drive people to seek information and support online, making this project’s digital presence particularly relevant.

**Methods:**

Building on methods developed in Parenting Science Gang, we conducted a co-production project within a private Facebook group from February to September 2021. Seventy individuals with depression and thirty researchers participated in discussions and chose a research question. Together, they designed and implemented a survey and focus groups exploring health-seeking behaviours and under-reporting of depressive episodes to GPs. Concurrently, a PhD student analysed UK Biobank data for discrepancies in depression reporting. We used multiple methods to evaluate the process and participant experiences.

**Co-produced research results:**

In the UK Biobank, 67% of individuals whose questionnaire responses indicated depression had no coded GP record of it. Similarly, 84% of DD participants reported visiting their GP for half or fewer of their depressive episodes. Key barriers included stigma, dissatisfaction with healthcare, and a preference for non-medical interventions. These alternatives were perceived as better addressing the root causes of depression.

**Evaluation results:**

Participants described the research process as rewarding, noting it broadened their understanding both of depression and their own experiences. Researchers valued the insights gained, but found the process demanding.

**Conclusions:**

This project demonstrates the value of incorporating lived experiences into mental health research through co-production. Lived experience participants contributed unique perspectives that differed from those of university researchers. By combining these insights with big data analysis, the project generated findings that better reflect real-life experiences, enhancing their applicability to personalised mental health care. Participants found the process to be ‘quasi-therapeutic’ and advocated for healthcare systems to adopt diverse, holistic treatments for depression. These findings support inclusive policy development and underscore the importance of epistemic justice and democratic knowledge production.

**Supplementary Information:**

The online version contains supplementary material available at 10.1186/s40900-026-00889-2.

## Abstract (plain language summary)

Depression affects many people, and researchers want to help. Data scientists use powerful computers to find patterns in large data sets. While data shows what has happened, it is not very good at working out the reasons behind it. People who have experienced depression can have insights that researchers might miss. So sometimes researchers and other people work together. This is called ‘co-produced research.’ We ran a project with data researchers and people who’d had depression. They had online conversations to learn about research and the science of depression. Together, they thought of many potential research questions. They explored these and chose: “Do people report all episodes of depression to their GP? And if not, why not?” Then, they decided which research methods to use. The data scientists checked the UK Biobank, a large health database. They found that 67% of depression episodes were missing from medical records. Had people not told their doctors? The lived experience participants carried out discussions and surveys to find out why. They found that people only visit the GP for about half of their episodes. Reasons for this included feeling hopeless, thinking the GP wouldn’t help, and not wanting antidepressants [medicine for treating depression]. The lived experience participants enjoyed the project. They appreciated the chance to talk to others and reflect on their experiences. They valued the opportunity to do real research, but sometimes found it stressful. The researchers learned more about patient perspectives but found the process hectic. We would like to do it again, but better.

**Supplementary Information:**

The online version contains supplementary material available at 10.1186/s40900-026-00889-2.

## Background

### Patient and public involvement in research

In this paper, we use the term Patient and Public involvement (PPI) as an umbrella to cover all collaborative efforts between the research community and individuals such as patients, carers, advocates, service users and community members [1]. Effective PPI values all contributions, ensuring people have a meaningful say in research and influence its outcomes, as set out in the UK Standards for Public Involvement [2]. PPI is often described as a spectrum or ladder, ranging from ‘Inform’ at the bottom (sharing information about the research), through ‘Consultation’ (seeking feedback), to ‘Involvement’ (integrating patients into the process), ‘Collaboration’ (partnering with patients), and culminating in ‘Empowerment’ at the top (with patients initiating and leading the research) [3]. This approach also supports epistemic justice [4], the idea that multiple perspectives must be recognised to understand what is going on, by ensuring that individuals with lived experience are acknowledged as legitimate knowers whose insights enrich and challenge dominant research paradigms [5].

Meaningfully incorporating lived experiences into research enhances depth and relevance by providing insights that might otherwise be missed [6]. It also ensures interventions are grounded in real-world contexts, ultimately producing more effective and sustainable results [7]. Furthermore, it fosters inclusivity, promotes equity and diversity, and enhances participants’ agency by involving them directly in the research process [8].

PPI in health and social care research has expanded significantly in recent years [9] rooted in the understanding that it is important, expected and possible across all research types [10]. Funders and policymakers promote PPI because it leads to both improved research outcomes and social justice [10]. Consequently, funding and policy levers have been introduced to support it [11, 12]. For example, UNESCO’s Open Science recommendations [13] emphasise the importance of community engagement. The EU’s Horizon 2020 programme funded 20 dedicated citizen science projects, including infrastructure to mainstream it [14]. Similarly, Research England’s £6 million Participatory Research Grants, launched in 2021, highlight substantial investments in this area [15]. Organisations such as the global charitable foundation, the Wellcome Trust [16] and the UK’s UKRI [11] and NIHR [3, 9] now mandate the inclusion of lived experience in all mental health research. The shift is also evident in research infrastructure, as mental health charities such as MQ [17] and UKRI’s Mental Health Platform [18] have incorporated PPI components. Additionally, the upcoming UK REF 2029 emphasises ‘Engagement and Impact’ with 25% of its weighting dedicated to these aspects [19].

However, some have criticised certain PPI efforts as shallow or tick-box exercises, targeting the ‘easiest to reach’ [20] and as failing to challenge or address power dynamics. Many researchers lack the knowledge, time or skills to involve patients or publics in significant or meaningful ways [21]. Furthermore, even the term PPI is contested [22], and the use of terminology varies greatly across the sector and between topic areas. This project aimed to respond to that challenge by offering a practical, supported model for deeper engagement.

### Role of big data in mental health

This project’s key focus was to bring together Mental Health Big Data researchers with people who have lived experience of depression. Big data plays a crucial role in understanding health trends. By combining large datasets from diverse sources, researchers can identify patterns and risk factors that might not be evident at smaller scales [23]. This is crucial for developing effective public health strategies [24]. However, the field has been slower than more clinical or social science areas of health research to incorporate PPI [25].

Nevertheless, while these large datasets can reveal correlations, they often lack the nuanced context required to understand causation [26]. They can tell us WHAT happened, but not always the reasons behind it. By contrast, PPI approaches can provide context and insights into the nuances of patient experiences, helping to illuminate why things are happening. Especially when these approaches are implemented well.

Researchers use various anonymised public data sources, including census data, electronic health records and prescription data [27]. These are complemented by volunteer contributions from cohorts such as the UK Biobank [28] and Generation Scotland [29], which provide additional data, including genetic information and self-reported health questionnaires. Combined, these data provide valuable insights into the prevalence and triggers of ill-health [30] and hold promise for personalised medicine approaches (such as those being advanced by the Wellcome-funded AMBER project [31]).

In this project, we piloted co-production with patients as a way to bring insight and context to the interpretation of Big Data results. We also sought to help focus Big Data’s attention on questions that are important to people experiencing depression. In this paper, we use the term co-production to mean that lived-experience participants and academic researchers worked together, as equal partners, through all stages of the project.

### Overview of depression

Major Depressive Disorder, often referred to simply as depression, is a mental health condition characterised by long-lasting, persistent low mood. Symptoms can include poor concentration, excessive guilt, hopelessness, thoughts of death, disrupted sleep, changes in appetite or weight and low energy [32]. It affects every aspect of a person’s life, including their long-term health, social well-being, family relationships, education, and career [33]. It can be categorised as mild, moderate or severe based on symptom severity and impact on everyday life. Depression is ranked by WHO as the single largest contributor to global disability (7.5% of all years lived with disability in 2015 [34]). In the UK, approximately 1 in 5 adults experience symptoms of depression at some point in their lives [35].

### Online engagement

Depression can lead to isolation, making daily activities and social interactions challenging [36]. This internal struggle often drives individuals to seek online support for information and peer connection [31], which has been shown to improve symptoms and enhance agency [37, 38]. However, the quality of online resources varies widely.

The lead author (SC) previously developed an online co-production research project for parents of young children [39] who may experience similar difficulties with leaving the house, personal care, and accessing meaningful adult interaction. Furthermore, at the time of this project, some COVID-19 restrictions were still in place [40], and many people were continuing to socialise and work remotely. The online model of engagement was a way to integrate lived experience into research, under the constraints of ongoing social distancing measures.

We recognise that online engagement excludes some people - for example, those without access to the internet. However, it is also more inclusive than in-person engagement for many people: those who are geographically isolated; parents of young children who don’t have babysitters; those with mobility issues; and people who are time-poor (attending an event also involves travel, parking, etc.).

Group interactions during the project were entirely text-based. (We had no video calls or similar). We made this decision because, in previous projects, we have observed several advantages of text-only interaction:

1. Everyone can type at the same time, and no one is blocked from contributing because other people are contributing at the same time. In voice or video calls, only one person can usually speak at a time, and it can be easy for a small number of louder or more confident participants to dominate the discussion.
2. Shyer or quieter participants tend to contribute more in text discussions. No one is judged for their accent or what they look or sound like.
3. Asynchronous conversation is much easier. Most activity in the groups was asynchronous, with people dipping in and out when it suited them. Although we had set times for some activities, we knew that any time we chose would not be suitable for some participants. With text, it’s easy to submit questions beforehand or add extra comments later.
4. Text is less data-heavy for those contributing on a phone, with a limited data plan. It doesn’t require cameras, microphones, or speakers.
5. Text doesn’t make a noise or require you to make a noise. You can interact without disturbing other people around you - on a bus, in a library, in a cafe, while your family are watching TV, while your baby is napping.
6. Text is familiar. Despite the Covid-inspired proliferation of video calls, text is still a very common means of communication for most people, including young people. WhatsApp, Snapchat, Discord chat, comment threads, games or on YouTube streams.
7. With limited staff time, text-based conversations were much easier to turn into blogposts and share outside the group, or with group members who couldn’t attend.

We acknowledge that there are groups for whom this is not the most appropriate mechanism. But every choice of medium excludes some and includes others. For a single short and constrained pilot project, this was the route we chose.

### The depression detectives project

The funding for this project was provided via data science research grants. The Patient and Public Involvement Co-ordinator employed on these grants (IB) had previously been involved in Nappy Science Gang [39] and Parenting Science Gang [39] and recognised an opportunity to bring the same co-production methodology into mental health data science.

This project was envisioned as a short pilot study (8 months) to investigate whether the techniques used in Parenting Science Gang (which lasted 2 years) were transferable to a different context. We wanted to provide proof of concept for meaningfully involving people with lived experience in big data research. Although similar in many respects to its predecessor, a key difference between the projects was the recruitment of researchers. For the Parenting Science Gang, the topics were selected by lay participants, after which a researcher was identified and invited to join the project. For Depression Detectives, the partnership with data science researchers from Edinburgh Neuroscience [an interdisciplinary, cross-college community at The University of Edinburgh] [41] ] was in place from the beginning. This lightly constrained the research direction but also provided opportunities for co-production from the outset.

The project was run as a partnership between Edinburgh Neuroscience, the Patient and Public Engagement Coordinator, and the social enterprise Science is People Ltd (SiP). Researchers and staff at the department participated actively, but most day-to-day facilitation was performed by project staff at SiP. One member of SiP staff has extensive experience in public engagement with science; one is a social science researcher with counselling expertise; and one has a background in communications and online community management. Several members of staff across both organisations had lived experience of depression and were open about their dual role and experiences in group discussions.

## Methods

### Ethical considerations

Ethical oversight was secured through the Edinburgh Medical School Ethics Committee (EMREC), with ethics permission reference 21-EMREC-004_SA2.

However, obtaining ethical clearance was challenging, as EMREC typically does not assess PPI/co-produced research projects.

Once the process began, it became clear that our participants were considered potentially vulnerable, necessitating the implementation of various safeguards (see Table 1). These included a lengthy Patient Information Sheet (see Additional Information 1), which, for data security reasons, had to be presented on a separate system (Jisc surveys). This created an additional, time-consuming sign-up step, which we suspect was a significant barrier for some potential participants. The ethics board also raised concerns about the use of a private Facebook group, specifically regarding accessibility and anonymity, as participants were required to have a Facebook account to participate, which is typically linked to their real identity. An exploration of these and other ethical issues is presented in Table 1.

**Table 1:** Summary of ethical issues, their relevance to the project and future implications

Topic	Description	Relevance	Now / Future
Ethical Clearance	The Ethics board to which we submitted was established to review research projects and lacked the capacity and expertise to review PPI/co-produced projects.	Some PPI projects may cause harm and should be considered from an ethical perspective. Many journals require evidence of ethical clearance before papers can be considered for publication.	The Edinburgh Medical School Research Ethics Committee (EMREC) have now produced a self-assessment tool to help staff determine when PPI projects may need to be considered by their ethics board [42] A new cross-college PPI ethics group is being established at The University of Edinburgh (personal communication with IB).
Facebook group	The activities took place within a private Facebook group that was ‘discoverable’, but no content could be viewed until an administrator verified that we had received a fully completed consent form. Posts did NOT appear on participants’ friends’ feeds (for example).	To join most Facebook groups, you just click a link and answer a few questions. The consent form created a barrier to joining the project and is likely to have deterred some participants (particularly those with lower literacy levels or greater scepticism about universities). It was important to several participants (and the ethics board) that their ‘Facebook friends’ did not view what was posted within the DD group.	The feasibility of a project like this depends on participants finding and entering the private group, and then being reminded of it via their Facebook feed. However, algorithms are continually evolving, which could affect group/post visibility. Reducing barriers to entry would potentially increase the chances of ‘Facebook Friends’ seeing posts.
Facebook Profile	Participants were required to have a Facebook account to participate in the project. However, we made it clear in the sign-up forms that we were only seeking individuals with existing accounts. We did not endorse the creation of new accounts to participate in the project.	Some people will have been excluded from the project because they lacked a Facebook account. However, for others, this made participation in the project much easier, as they visited the site regularly anyway. Most people use their real name to set up a Facebook profile, so they give away their real-world identity within the group. Our Patient information sheet and a pinned post reminded participants that they may not be fully anonymous to others when participating in the group and that Facebook may also be collecting data, based on their participation.	Other platforms could perform a similar function in future projects, but each choice comes with pros and cons.
Patient information sheet	Before people could join the Facebook group, they had to read and acknowledge a long Patient Information Sheet.	This was not the most appropriate format/length/style for this project.	Sector-wide conversations are ongoing around additions/alternatives to long patient information sheets.
Ethics boards are risk-averse	Our participants, by definition, had self-disclosed lived experience of depression, so were potentially vulnerable.	People could only join if they did not consider themselves currently depressed. We had to collect participants’ offline contact details and request an emergency contact (a trusted friend or relative). We encouraged them to complete a Wellbeing and Safety Plan at the start of the project, in which they could consider their own triggers and the steps they would take if they began to experience difficulties. We also provided a list of resources for assistance (phone numbers and websites). We had a qualified counsellor as part of the project team, and a Clinical Psychiatrist was available to discuss any concerns [this scenario never arose].	In Beange et al. (2024) [43], current Wellcome-funded project (AMBER), the lived advisory group discussed safeguarding without infantilizing. They chose to have a safeguarding protocol with optional individual safeguarding plan. Sector wide discussions are ongoing around how to safeguard our lived experience participants.
Moving information between platforms	We moved Q&A transcripts from the closed Facebook group to the publicly accessible blog. We also summarised other discussions/information for the blog.	All names and identifying information were removed from transcripts and discussions before they were uploaded or summarised for the publicly accessible blog / used in any public way. At least 2 people checked each item before it was uploaded.	
Ethics need details of the research question & method.	We had to resubmit/update the ethics section midway through the project, once we had finalised our research question and methodology.	This introduced a delay of several weeks in the middle of the project, which lived experience participants questioned. Requesting a quick turnaround by ethics meant that we were restricted to asking our survey/focus group questions only to people already in the group (limiting our sample size).	The new PPI ethics group at The University of Edinburgh will consider and create policies on how to deal with this type of co-produced project in the future.

Nevertheless, this project (amongst others) has stimulated ongoing efforts at the University of Edinburgh to improve ethical oversight for PPI initiatives, including the establishment of a dedicated ethics group (in progress).

### Setting

As project organisers, we chose to conduct the majority of the project in a private Facebook group. At the time, most social media users were on Facebook (83%) [44], and 61% of UK adults visited it daily [45]. However, it’s worth noting that the social media landscape has since evolved, with platforms such as TikTok, Snapchat, and Instagram becoming increasingly popular, particularly among younger generations [46]. The predecessors to this project (Parenting Science Gang [39] and Nappy Science Gang [47]) were also hosted on Facebook, benefiting from the vibrant community of parents and new mothers that could be found there.

To give the project a public presence, a project blog was also set up to host project details, progress updates, and anonymised Q&A transcripts. This blog served many useful overlapping purposes. It improved project transparency and provided content to share when recruiting public participants, briefing researchers, or approaching other organisations to discuss the project. A key part of the blog was the (anonymised) Q&A transcripts, which were then easy to share (e.g., in the Facebook group, elsewhere on Facebook and to other social media platforms). As a project legacy, the blog serves as an archive of posts on various aspects of depression, which remain accessible to a general audience. The blog also makes it easier for other interested parties to see the detailed steps of how the project worked, in an alternative way from reading this paper.

### Process

**Fig. 1:**
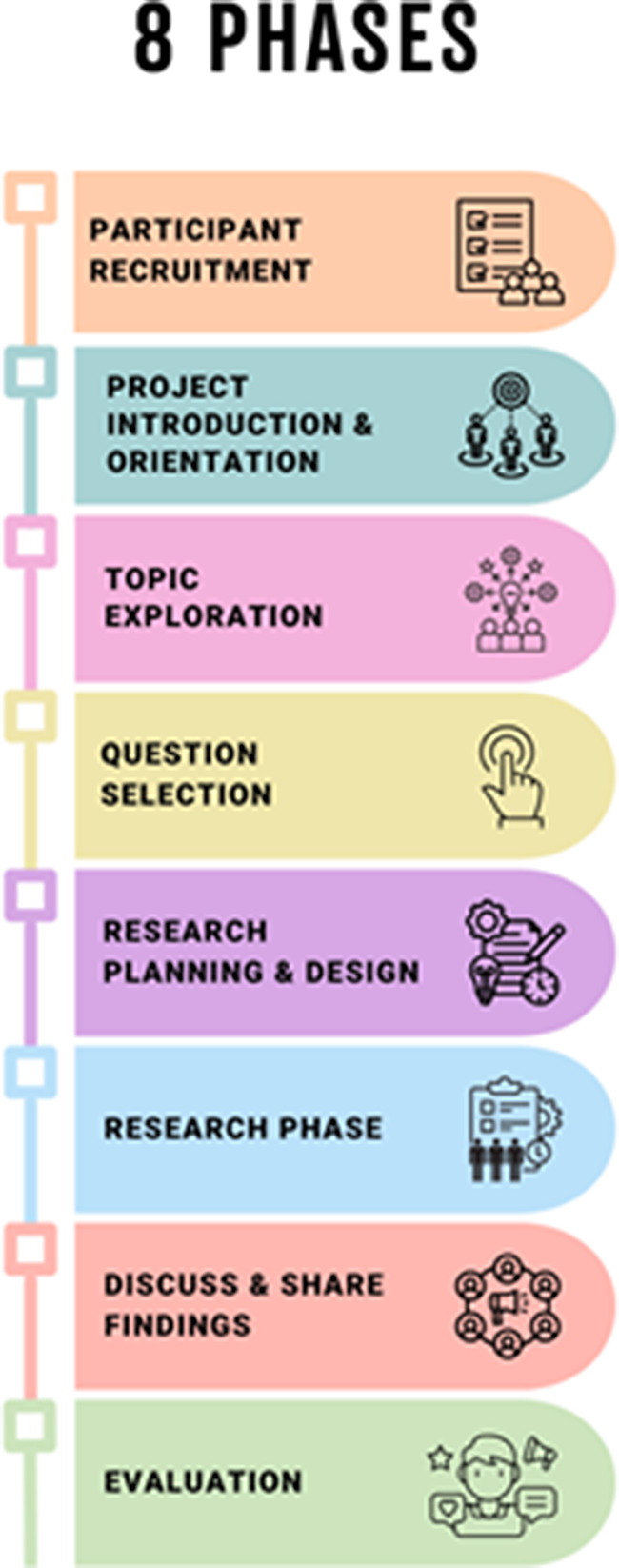
The 8 phases of the project

The project used an 8-stage process (see Fig. 1).

### Phase 1: Participant recruitment

We recruited lived experience participants [from this point forward referred to as ‘participants’] through various channels, focusing on those 18 and over who had experienced depression, but who were not currently depressed or in crisis. Although we aimed to recruit Scottish participants for this pilot, we were open to participants from any location.

Recruitment channels included emails distributed via Scottish/UK mental health charities/organisations, posts in relevant mental health Facebook groups, mailing lists, participants from previous projects, personal networks, and social media (see Table 2). By using multiple channels, we aimed to get a broader audience. However, despite our efforts, in a small, proof-of-concept, exploratory pilot such as this, there was no way to ensure we reached every group, and we acknowledge that this project would not appeal to everyone. Participants were also self-selecting, so may not be typical of the entire population of people who experience depression. They are likely to be more proactive about their mental health and more confident communicating in English, by text. They also needed to have a Facebook account.

Sign-up forms (see Additional Information 1) included questions about participants’ views on mental health research and their confidence in finding, evaluating, and understanding scientific information, as well as in expressing disagreement with healthcare providers, which aided subsequent evaluation.

Participants were not financially compensated. Our experience with Parenting Science Gang [39] (where we had 2,600 participant volunteers) suggested that participants felt they derived a lot of personal value from their participation in the project, and did not perceive it as inequitable. We also valued participants’ ability to dip in and out as their interests and availability changed, while still making meaningful contributions to the project. It also meant that participant numbers did not need to be capped.

Initially, researcher recruitment was predominantly from the research group that was funding this pilot. Their PI (Principal Investigator) had agreed to the pilot project, but other individual researchers did not necessarily know the details. Therefore, internal communications and “coffee morning” sessions were used to help researchers learn more about the project. Additional researchers were subsequently recruited from across Edinburgh Neuroscience, The University of Edinburgh, and beyond, in response to the topic requests and needs of the lived-experience participants.

**Table 2:** Recruitment Methods

Lived Experience Participants	Researchers
Posts in relevant mental health groups on Facebook	Internal communications and Edinburgh Neuroscience mailing lists
Emails sent out via Scottish / UK organisations (e.g., mental health charities)	‘Coffee’ Zoom drop-in sessions for University of Edinburgh staff.
Previous PPI activity participants	Targeted recruitment later in the project (predominantly via Edinburgh Neuroscience), in response to the topic requests of the lived experience participants.
Participants from similar projects (Parenting Science Gang, Nappy Science Gang)	
University of Edinburgh community and PPI email lists	
Team members’ and researchers’ Twitter, Facebook, LinkedIn, and email accounts.	

### Phase 2: Project introduction & orientation

**Fig. 2:**
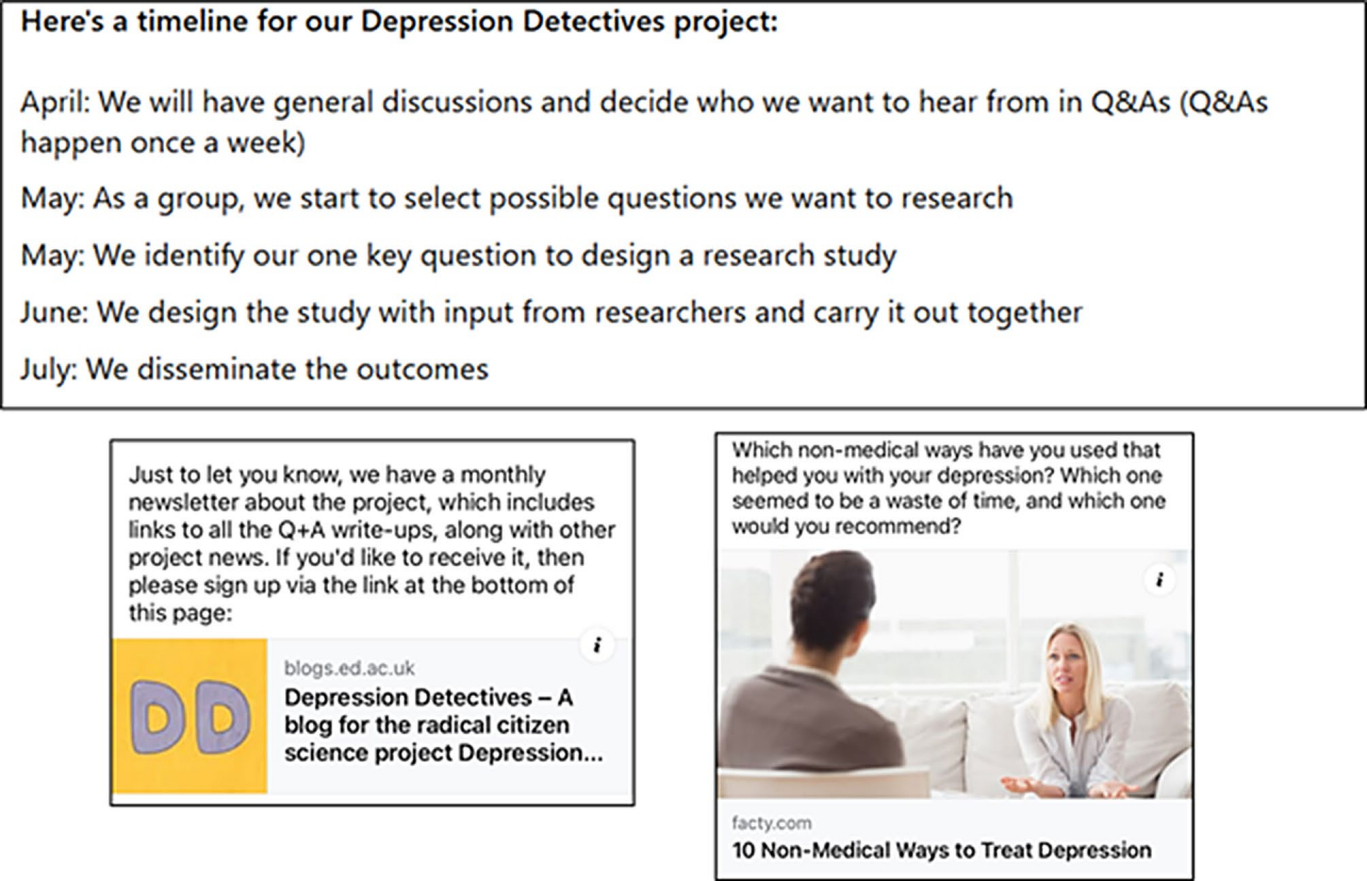
Example Facebook posts during the introductory phase

This phase involved getting to know each other and the project, as well as establishing our interaction style (see Fig. 2). We posted frequently in the group to clarify our goals, opportunities, and, importantly, our constraints. Post types included:

- explanation of co-produced research,
- overviews of previous projects,
- participant roles and expectations,
- survey reminders,
- tips on notification settings.

We repeated the same topics several times to ensure they were seen. In previous projects, participants often said, “We wish we’d known X,” despite multiple posts on that very topic. We also asked participants to help us revise our provisional group rules (used in previous projects) and to suggest any new rules they thought would help ensure productive and collaborative discussions.

Our tone was chatty and encouraging, prompting participants to interact, which, of course, was the project’s primary purpose. Engagement, especially comments, also boosts the Facebook algorithm and improves the visibility of posts. Nevertheless, based on prior experience, we knew it was essential to use a variety of post types to guide participants from lurking to active participation. Polls were used for quick, simple interactions (e.g., best time for Q&A session, pick a topic from a list), whereas simple open-answer threads were used for quick text-based answers (e.g., introduce yourself, what topics you are most interested in). We combined these with more topic-based discussions, which featured content such as articles (journal articles, official medical guidelines, popular science and news articles), and creative content (podcasts, videos, art exhibitions, film festivals) that could be discussed from a variety of angles (see Fig. 2).

This phase also included our first Q&As (question and answer sessions). These were a key part of our method, involving a researcher coming online at a specific time, briefly introducing themselves and their topic, and then providing text-based (typed) answers to our participants’ questions. After a trial of conducting these in the main Facebook group, we decided to move them to separate Facebook groups, making them more of an ‘event’ and avoiding cluttering up the main space. Initial sessions explored perspectives on depression, including clinical psychology, brain imaging, social anthropology, and treatment options.

### Phase 3: Topic exploration

This phase focused on expanding topics and generating new ideas. We assumed participants understood our goals but continued to share reminders and overview links. We arranged additional specialised Q&A Sessions (e.g., depression and sleep, pregnancy and childbirth, COVID) to introduce participants to available researchers and potential research methods. Before each Q&A, we shared posts about the topic and the researcher, encouraging discussion and collecting questions in advance from those unable to attend. At the start of the Q&A, the central project team helped people get started in the group by asking basic questions. They then actively took part, sharing their own (varied) views and experiences. They modelled asking clarifying (‘stupid’) questions and saying ‘I don’t understand’, as well as ensuring comprehensive coverage of the topic (the what/how/why of the research). After each Q&A, we organised and anonymised the transcript, posting it on the project blog as a discussion resource for the group, a dissemination tool for the researcher and as an ‘advert’ on social media to attract new members. All participants were aware of the project blog and encouraged to use it as a resource to share with others.

In addition, we continued to facilitate online discussions, encouraging participants to begin suggesting research questions. That is, questions to structure an original piece of research around.

### Phase 4: Question selection

This was a narrowing phase, after the expansiveness of the question-generating phase. During this phase, the group collaborated with researchers to select a question and start designing a project that fit within the budget, time, and practical constraints.

The group generated 59 potential research questions. We listed them all in a single post, in the exact words of the questioner, and then repeated them below the post, each as a separate comment. This allowed a separate ‘sub-thread’ to form under each question. Throughout the week, we encouraged active discussion and shared resources, such as guidance on conducting Google Scholar searches and understanding different types of scientific studies. We also kept reiterating our timelines, where we were in the process and the next steps. Members then had a further week to vote for their top three choices, using the ‘reaction’ buttons. This voting method (range voting) is used to identify the solution most palatable to the greatest number of voters.

The vote produced a top-ten list (see Additional Information 2). Daily ‘spotlight’ threads followed, where we discussed each of the top ten questions in turn, exploring why they interested us and any links to previous discussions. We also discussed the implications of the research, how it might be researched (methods), the resources and researchers available, and what might be feasible within our timescales and logistics. (Please see the discussion section for more on this process and why it was designed like this.) The quietest spotlight thread had 16 comments, and the busiest one had 55 comments.

**Fig. 3:**
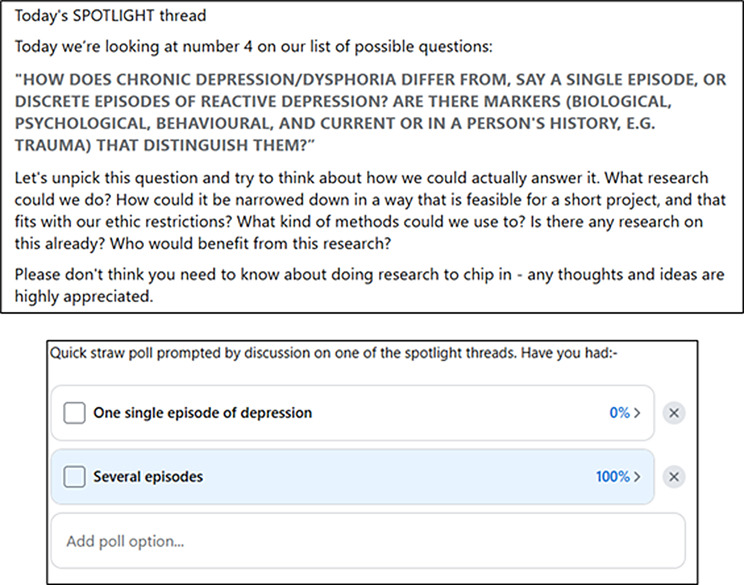
Example spotlight thread and follow-on poll

After ten days of ‘spotlights’ and other conversations alongside those threads (see Fig. 3), we then opened voting on the top ten, using the same process as above (post with each question as a comment and range voting via the ‘react’ buttons). The winning question was:

“How does chronic depression/dysphoria differ from a single episode or reactive depression? Are there markers (biological, psychological, behavioural, historical, e.g., trauma) that distinguish them?”

### Phase 5: Research planning and design

In this phase, we explored the chosen research question from as many angles as possible. Participants shared their interests and expectations, and we discussed terms and words related to the question, comparing scientific and personal definitions. We shared relevant papers and discussed the findings, exploring their implications for us and this project.

A group poll revealed no one had experienced a single episode of depression, sparking several discussions on whether multiple episodes are more common or if people who experienced a single episode were less likely to ‘identify’ as a person with depression and join a project like this. We also debated the term “episode,” with several members expressing that the word didn’t characterise their experience at all. (See results section for more details and quotes).

We held a Q&A with three data researchers on using cohort study databases, and another with a research consultant to explore non-data science options and discuss what participants wanted to gain from the research. These sessions enriched our discussions and encouraged researchers to come into the Facebook group to address participants’ queries.

This phase mirrored the two previous ones, expanding outward in all directions and then refining ideas until the focus narrowed to two key research strands. The revised research question became: “Do people report all episodes of depression to their GP? And if not, why not?” (GP = General Practitioner - community-based primary care doctor in the UK).

The plan evolved into two parts:

- **Data Science Research**: Conducted by a PhD student using UK Biobank data [28]. She compared self-reporting of depression (sign-up questions and completion of standardised depression screening questionnaires) to codes relating to depression in Electronic (GP) Health Records.
- **Social Science Research**: Surveys and Focus Groups led by the participants to explore experiences of ‘depression episodes’ and why, how, and where people seek help.

### Phase 6: The research phase

This phase was when the research participants co-produced and conducted specific research activities. Facilitated by social science researchers, participants crafted prompt questions for focus groups (targeted discussion threads). These included:

- What are “episodes” of depression for you? Does speaking of “episodes” make sense to you?
- What went on for you when you tried to decide what kind of help to seek? If you went to your GP, what made you decide to go? If not, did you consider going? Why did you decide not to?
- What has your experience with primary care staff (doctors, but also receptionists) been like? What worked, and what would you like to be different?
- What kind of help would you like to be offered when experiencing depression? What would be most helpful to you, and why? Could all of this be offered by GPs? What has been offered to you but was not helpful?

At this point in the project, we focused primarily on these targeted discussion threads [focus group questions] and used numerous reminders to encourage participation.

Responses from these discussions formed the basis of a co-produced survey (see Additional Information 3). The group wanted to distribute this widely, but that would have required an ethics amendment, which would have delayed the process by months, making it infeasible (we had only 2 months left, which could not be extended due to funding restrictions). We were therefore limited to the group’s ~ 100 members. This is a common challenge in co-produced research, where the question and method do not exist at the start. A completion deadline for the survey was set, and a staff member analysed the combined focus group and survey findings.

The “big data” strand, managed by a PhD student, was less interactive. For ethical reasons, group members were unable to assist with the analysis of UK Biobank data [28]. To access the data and participate fully in the analysis, group members would have required training in data privacy, an academic email address, and completion of the training required to gain ‘approved researcher status’ with UK Biobank. Combined with the necessary practical analysis skills, this would have involved substantial time and resources that were not available for this pilot project. However, we were able to discuss aspects like the 96 different codes that are available to GPs to record depression; the measures that are used, such as the Patient Health Questionnaire (PHQ-9) screening questionnaire [39] and the questions that are asked in UK Biobank questionnaires (such as “How many periods did you have in your life lasting two or more weeks where you felt like this?”). Otherwise, the student worked independently on the data analysis and later presented her findings to the group.

### Phase 7: Discuss and share findings

We posted the results from both research strands to the group (survey and focus group results and PhD student Biobank research findings). We did this both as summary reports and as individual graphs and charts to encourage engagement and discussion. Group members discussed whether they found anything surprising about the findings, and how the overall findings compared to their own experience.

We also asked the group to consider dissemination - who they wanted to inform about this research, and what they wanted to convey to them. The group identified several key audiences:

- GPs and healthcare professionals.
- Individuals with lived experience of depression.
- Policy-makers.
- Data science researchers.

A staff member created infographics for each group, highlighting key messages, and we asked participants to help share them.

### Phase 8: Evaluation - public and researcher

#### Survey for lived experience participants

The survey consisted of 18 questions, a mix of qualitative and quantitative items (See Additional Information 4). It requested some demographic data, their opinions on scientific research, and their approach to managing their own mental health. Additionally, it asked about the perceived usefulness of various activities within the project and overall satisfaction with their experience.

#### Semi-structured interviews with lived experience participants

Four participants were interviewed by one of the co-authors and project staff members (CK), who is an experienced qualitative researcher. The participants were selected by the project team to represent a spectrum of activities within the project, based on their posting and commenting in the group and Q&A Sessions. This was to provide insight into different experiences of the project and different levels of involvement. Everyone who was invited agreed to be interviewed. Interviews lasted approximately 45 min and were recorded on Microsoft Teams. The interviewer transcribed them and analysed them thematically. The prompt questions were about:

- Their background and previous experience in research.
- Their motivations for getting involved in this project.
- Their experiences of the project - both good and bad.
- What they thought they had got out of the project - learning, feelings about science, etc.

#### Survey for researchers

The survey for researchers consisted of 8 questions, mostly open-ended, asking about their experiences of participating in the project and its impact on them (See additional information 4). It also asked them to describe, in their own words, the project’s purpose and their role within it, to gain a deeper understanding of their expectations and views on public engagement and co-produced research.

#### Semi-structured interviews with researchers

As the previous Parenting Science Gang project [39] had focused its evaluation primarily on the lived experience participants; we wanted to ensure that we had sufficient process evaluation inputs from researchers for this project. We therefore selected five researchers to be interviewed. The project team selected these researchers to represent a spectrum of activity in the project, as we wanted a range of views and experiences. A team member invited them, and all agreed to be interviewed. As CK (who did lived experience interviews) did not have the capacity to conduct these interviews, we selected the same independent researcher who assisted in evaluating Parenting Science Gang. Each interview took approximately 45 min and was recorded on Zoom; it was then transcribed and analysed by the interviewer. The prompt questions were about:

- Their expectations of the project.
- Their role as researchers in co-produced research.
- Their experiences of the project.

SC oversaw the entire evaluation process and was responsible for bringing the different elements together.

## Results

### Recruitment

We recruited just over 100 members to the Facebook group. Approximately 30 of these were researchers. The numbers were lower at the start of the project and grew as the project progressed. Unfortunately, demographic data were deleted before they could be analysed.

### Question generation and selection

In phases 2–4, the group held 9 online Q&A sessions, with 14 researchers (some participated in the Q&A alone, while others requested to be accompanied by 2 or 3 colleagues).

The group generated 59 possible research questions, narrowed them down to a top ten (See Additional Information 2) and then chose their number one question:

*How does chronic depression/dysphoria differ from*,* say*,* a single episode*,* or discrete episodes of reactive depression? Are there markers (biological*,* psychological*,* behavioural*,* and current or in a person’s history*, e.g.,* trauma) that distinguish them?*

### Co-produced research design

As this question was too big to be tackled in a small project, after much discussion, the group reformulated it to: “Do people report all episodes of depression to their GP? And if not, why not?”

After discussing potential methodologies, the group split the research into two main areas:

- Data science research to examine whether all episodes of depression are documented in GP records.
- Surveys and focus groups to explore experiences, reasons for visiting or avoiding the GP, and alternative sources of help.

### Co-produced research results

#### Data science results

**Fig. 4:**
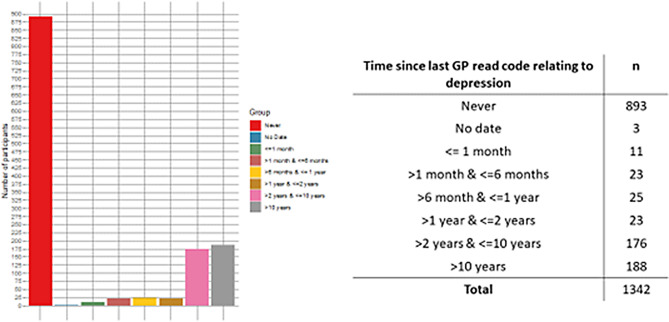
Time since last GP read code referring to depression. Firstly, we selected UK Biobank volunteers who were currently depressed at the time of completing the mental health questionnaire according to the CIDI and PHQ9 definition. The CIDI-SF (Composite International Diagnostic Interview – Short Form = lifetime measure, i.e., it measures if an individual is likely to have been depressed in their lifetime. PHQ9 looks at symptoms of depression in the previous 2 weeks. There were 123,040 UK Biobank volunteers who identified as not currently depressed and 2,907 who identified as depressed. Secondly, we identified UK Biobank volunteers who were depressed (according to the mental health questionnaire) and who had consented to giving access to their GP records. 1,342 volunteers identified as depressed AND had provided access to their GP records. Thirdly, we identified 96 read codes that related to depression [50]. We looked for these read codes in the GP records of the consenting UK biobank volunteers]

In the UK Biobank study, 500,000 participants were initially assessed, of whom 157,000 completed a mental health questionnaire. Of these, 2,907 were identified as currently/previously depressed using CIDI-SF [48] and PHQ-9 assessments [49]. Among 1,342 participants consenting to share GP records, 67% retrospectively self-reported a depression diagnosis but had no GP-documented depression diagnosis (See Fig. 4). This suggests significant underreporting of depression compared to non-depressive conditions, as only 1% lacked GP records for other issues. The findings emphasise potential gaps in GP consultations for depression (See discussion for limitations of the data science method).

#### Results arising from the participant focus group and survey combined

Ten participants took part in focus groups, based on the chosen question. The focus groups were conducted across four Facebook discussion threads. All names have been changed.

The focus group discussions then formed the basis of the co-produced survey (See Additional Information 3), which was completed by 26 people. As the survey was completed anonymously, it is unclear whether the focus groups and survey participants overlapped.

The survey results provided quantitative data (numbers and graphs). However, because they are based on the subjective experiences of 26 people, we consider the numbers less important than the underlying messages. We therefore present the survey results below, alongside a combination of free-text responses and focus group themes to bring these findings to life. Although 26 people completed the survey, respondents did not always answer every question, so some denominators are smaller than 26.

#### Episodes

The term “episodes” was a point of contention among focus group and survey participants. Some found that it accurately described their experience, while others disagreed.

**Fig. 5:**
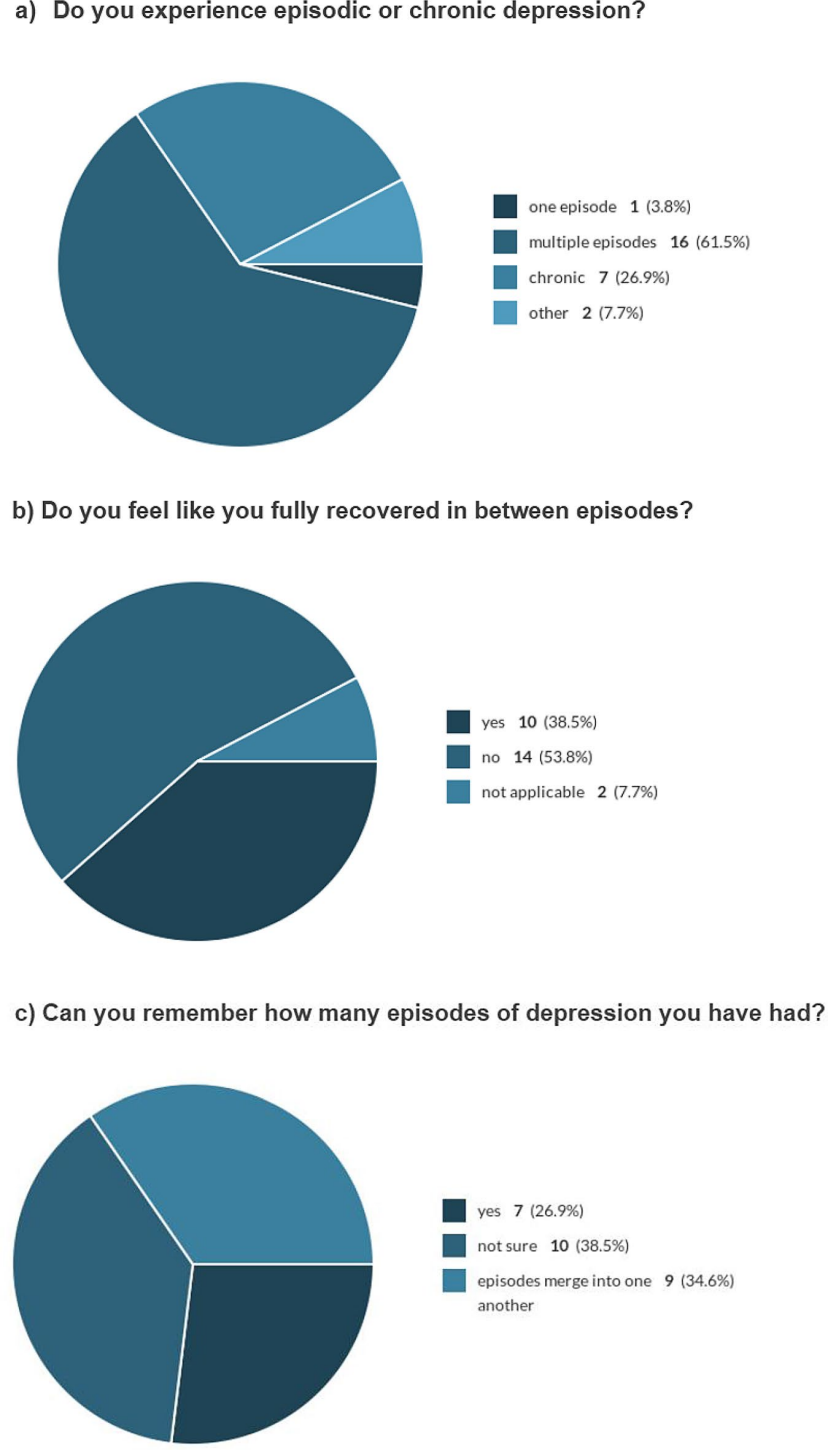
Episodes of depression (survey results)

When asked in the survey if they experienced episodic or chronic depression, only 1 person (out of 26) selected ‘one episode’. 16/26 people selected multiple episodes, 7 indicated it was a chronic condition and 2 people selected ‘other’ (See Fig. 5a). When asked to expand on ‘other’, one person described having experienced depression from childhood, but now in their 40s, considered themselves ‘cured’; and the other felt that their episodes had no “clear beginnings or ends” (survey comment).

When asked about recovery between episodes, 14 out of 26 survey respondents said they did not fully recover. 10/26 considered that they did, and 2 felt it was ‘not applicable’ (see Fig. 5b). Asked if they could remember how many episodes they have had, 7/26 said yes, 10/26 selected ‘not sure’, and 9 felt ‘episodes merge into one another’ (See Fig. 5c). When asked to approximate the number of episodes they can remember, the number ranged from 1 to 50 episodes (21 responses), with no clear consensus (see Table 3).

**Table 3:** Approximate number of episodes you can remember (survey result)

Number of Episodes (free text)	Number of participants (out of 21)
1	1
3	3
5	1
6	4
7	2
8	1
10	1
12	4
20	1
30	1
50	1

In the focus group, several participants disliked the term ‘episodes’, arguing that this term prevented doctors from recognising it as a “chronic condition that requires ongoing treatment/medication”. As one stated, “Depression is not something that goes away if you just wait it out.”

#### Help-seeking behaviours

**Fig. 6:**
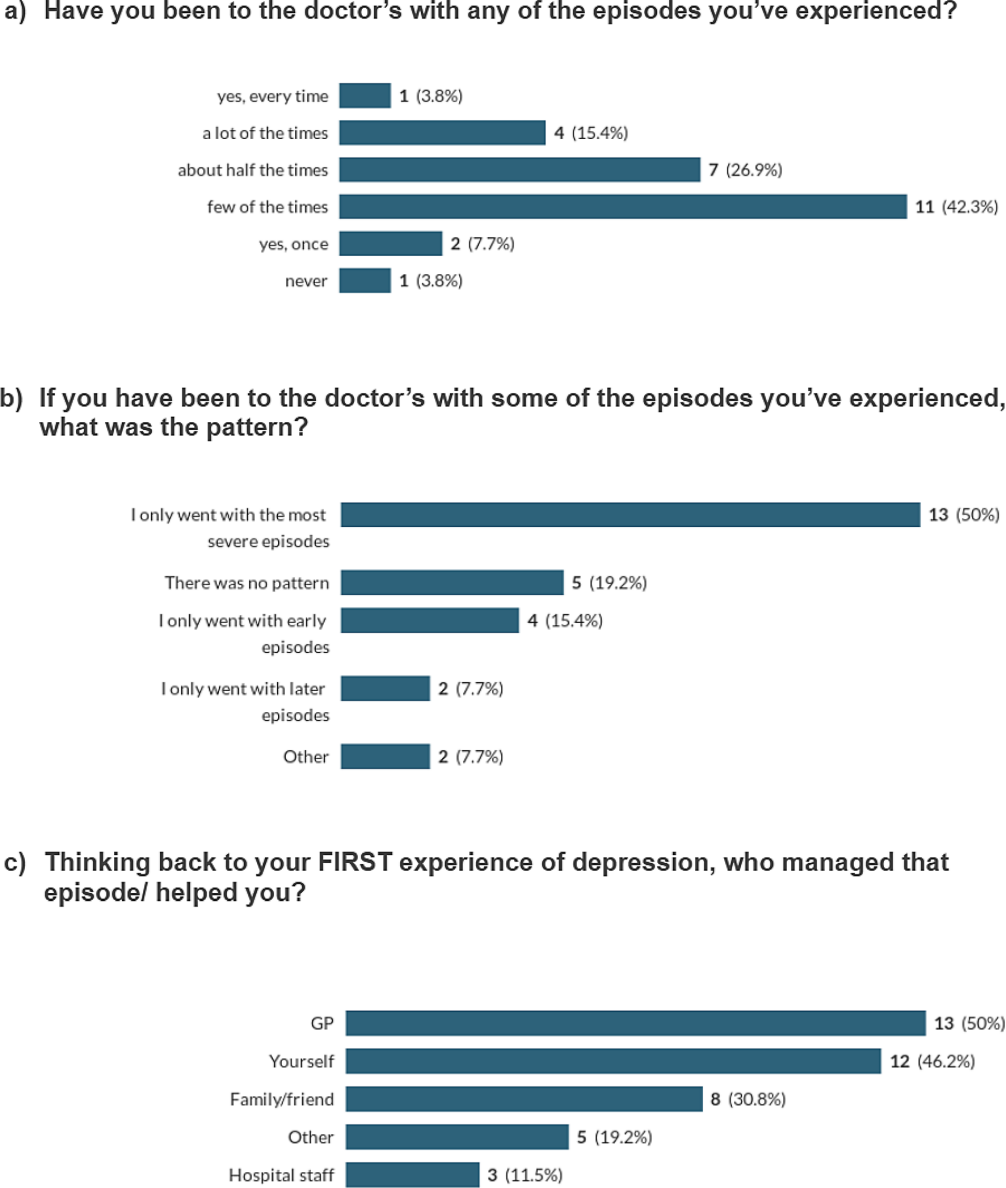
Episodes of depression and the doctor

When asked in the survey if they had been to the doctors with any of their episodes, only one (out of 26) responded ‘yes, every time’. 4 people selected ‘a lot of the times’, 7 chose ‘about half the times’, 11 ‘a few of the times’, 2 responded ‘once’ and one person had never been to their doctor (see Fig. 6a). When asked about the pattern of doctors’ visits, 13 (out of 26) replied that they ‘only went with the most severe episodes’. 5 reported ‘no pattern’, 4 ‘only with early episodes’, 2 ‘only with later episodes’. 2 people selected ‘other’ (see Fig. 6b). When asked to expand on the ‘other’ option, one person said, “I don’t have episodes. It is a chronic condition”, and the other said, “When I knew how to access mental health support elsewhere (e.g., privately or through work), then I didn’t go to the GP.” When more specifically asked if they had episodes of depression that they didn’t go to the doctor with, 25/26 said yes. Only one person said ‘no’.

When survey participants were asked to think about their first experience of depression (and select all that applied), GPs did come out as the top answer (13/41 responses, from 26 people). 12 people also chose ‘yourself’. 8 people selected family and friends (see Fig. 6c). Some survey respondents mentioned hospital staff (3 responses). ‘Other’ responses included university staff (2 responses), counselling (1 response) and one person responded that they were “not diagnosed until ten years later”.

In the focus group, participants described that despair, lack of coping, or running out of self-help options often drove them to seek help. Encouragement from others was frequently needed, as approaching a GP felt daunting. Furthermore, many found it hard to speak with receptionists, feeling self-conscious and unsure of what to say.

Our Focus groups identified 38 reasons for not visiting a GP. These 38 reasons were then offered in the survey for people to select all that applied to them. The top 3 reasons people chose were:

- Didn’t think I was depressed ‘enough’ to justify going to doctor (18/26).
- I just thought what I felt was part of who I am, rather than it being an illness (15/26).
- Hoped it would pass by itself (12/26).

Most of the other reasons given fell into two main themes:

- Feeling negative about themselves or about life. For example:

- I was ashamed about not being able to cope (11/26).
- I didn’t feel any hope that anything could make it better, at the time (9/26).
- I didn’t feel worthy of medical treatment and getting better (7/26).

b) Feeling negative about medical professionals or the options they could offer. For example:

- I had negative past experience with GPs who was unhelpful (9/26).
- The GP would not be able to give me enough time to explore what is going on and what I need (11/26).
- I thought other options not on offer from GP would be more useful (11/26).

**Fig. 7:**
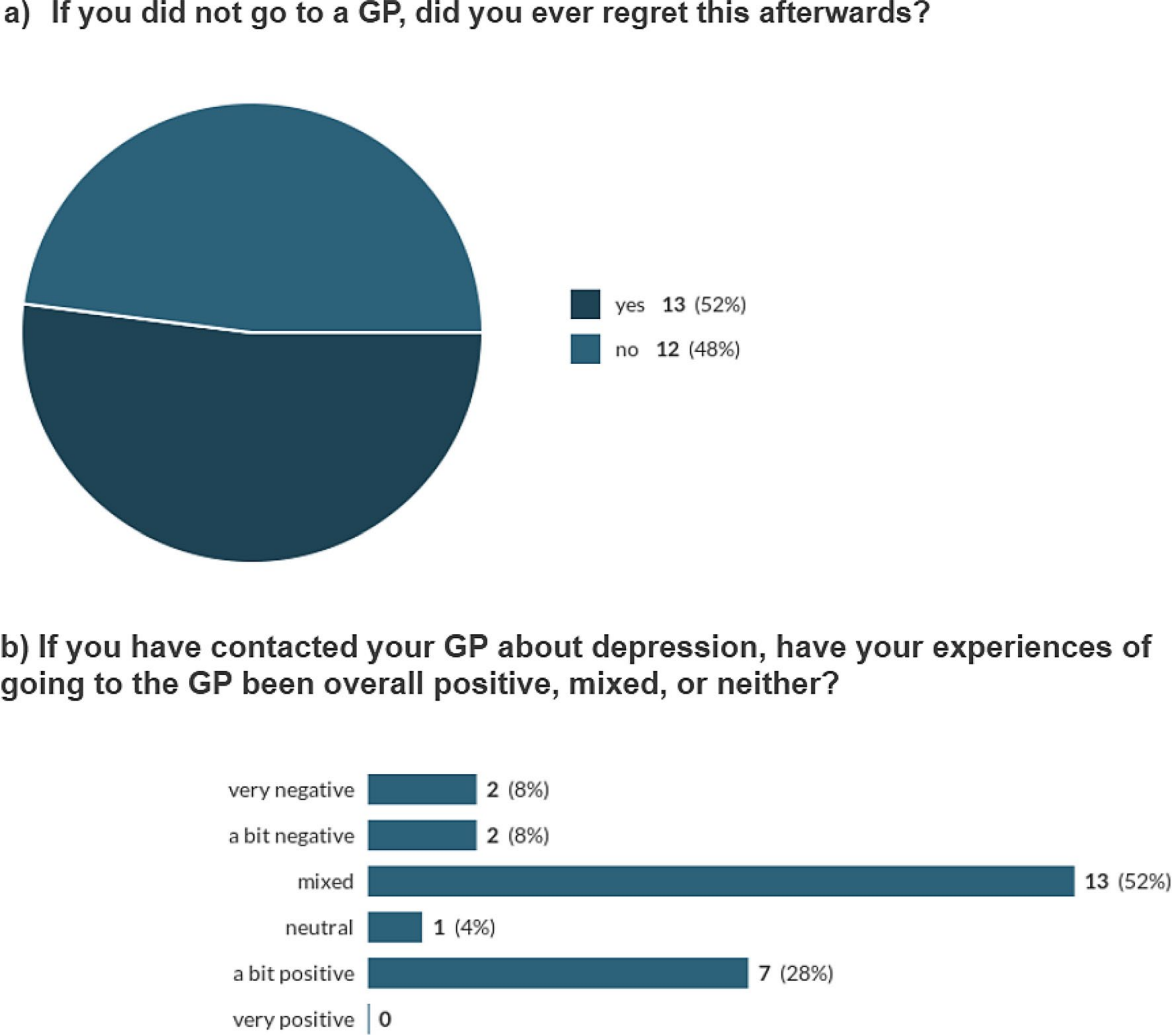
Experiences with GP

Nevertheless, with hindsight, over half of our survey respondents (13/25) regretted not consulting a GP (See Fig. 7a).

Most survey participants described their experiences with GPs as mixed (13/25), with the next most common response being “a bit positive” (7/25). No one selected ‘very positive’.

2 selected ‘very negative’ and 2 selected ‘a bit negative’. One person chose ‘neutral’ (See Fig. 7b).

Common complaints described by focus group contributors included feeling dismissed, rushed or not listened to. Some felt the GP was unkind or unempathetic. *“That one negative experience always stays with me*,* though*,* I am terrified of having to see someone who is similar in the future.”* However, people understood that some of this was related to the constraints under which the GP was working. *“I do think there are some GPs who are simply prejudiced about mental illness*,* but I think the main problem is the overall system they work in.”*

#### Alternative care options

Both survey and focus group respondents expressed disappointment over the limited care options available from their GP, with medication and short CBT (cognitive behavioural therapy) courses often seen as inadequate. Many people felt that medication, or short courses of CBT, were ‘just sticking plasters’ that didn’t address the root causes of their distress.

*My mental health has never been looked at holistically*,* only ever as a set of symptoms at a specific point in time (the appointment). (Focus group respondent)*

**Fig. 8:**
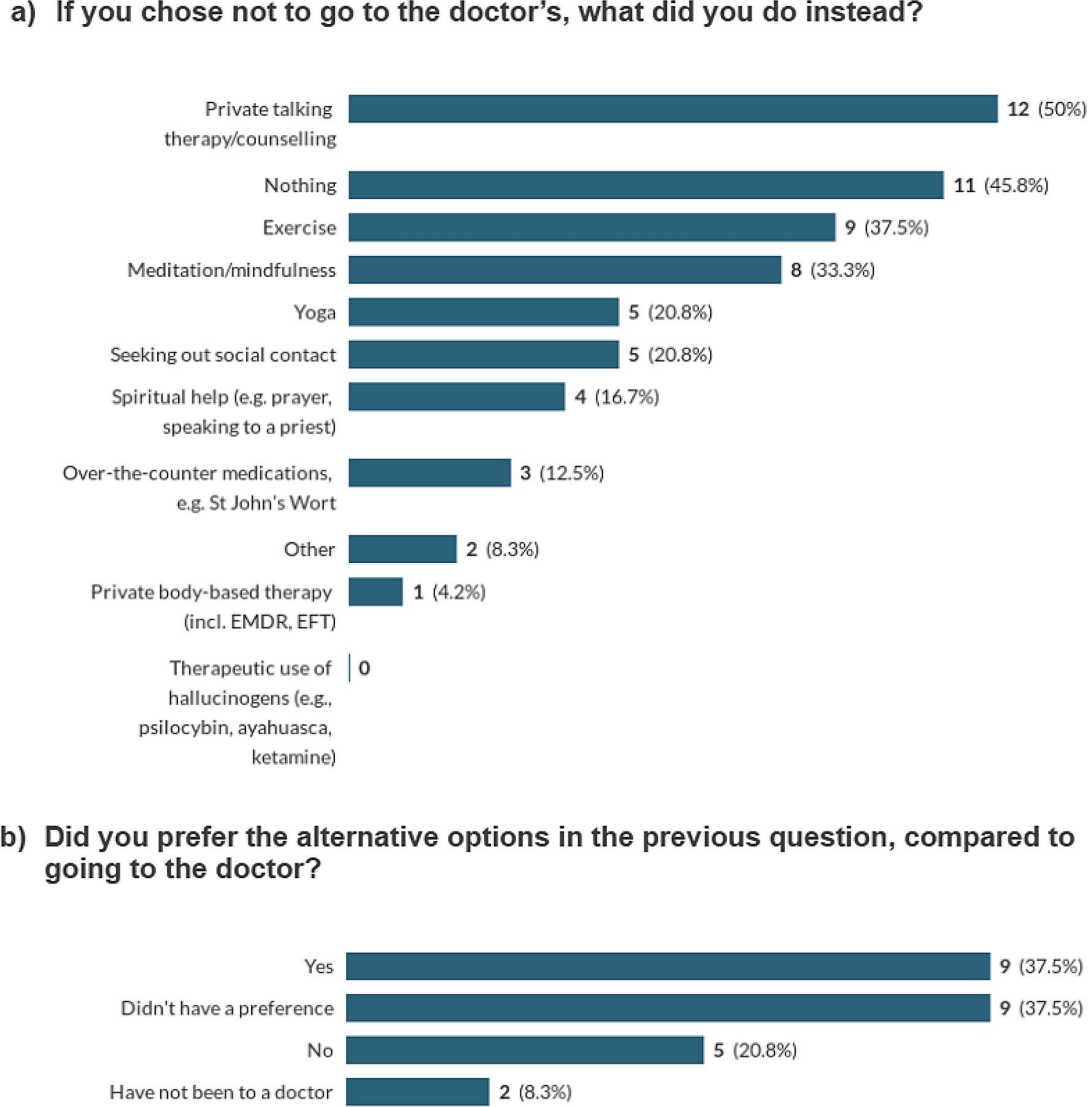
Alternatives to visiting the doctor

Many survey respondents sought private therapy (12/60 responses, from 24 people who answered this question; NB: they could select multiple answers). However, the focus group respondents told us that seeking out a good quality therapist, while depressed, was challenging. Alternative options included exercise (9/60) mindfulness/meditation (8/60), yoga and exercise (5/60 responses), seeking out social contact (5/60), spiritual help e.g., prayer or speaking to a priest (4/60), over the counter medications e.g., St John’s Wort (3/60), Private body-based therapy including EMDR, EFT (1/60) (See Fig. 8a). Respondents also listed ‘learning positive psychology’ and ‘self-help books’ in the ‘other’ category. 9/25 survey respondents preferred the ‘alternative options, compared to going to a doctor’. Yet another 9/25 said they ‘didn’t have a preference’ and 5/25 responded ‘no’ they would prefer to see a doctor. 2 stated that they have not been to a doctor (see Fig. 8b).

Focus group participants wished that all of these ‘alternative’ services were available through their GP, or that support was provided to identify the right help.

#### Key points for dissemination

**Fig. 9:**
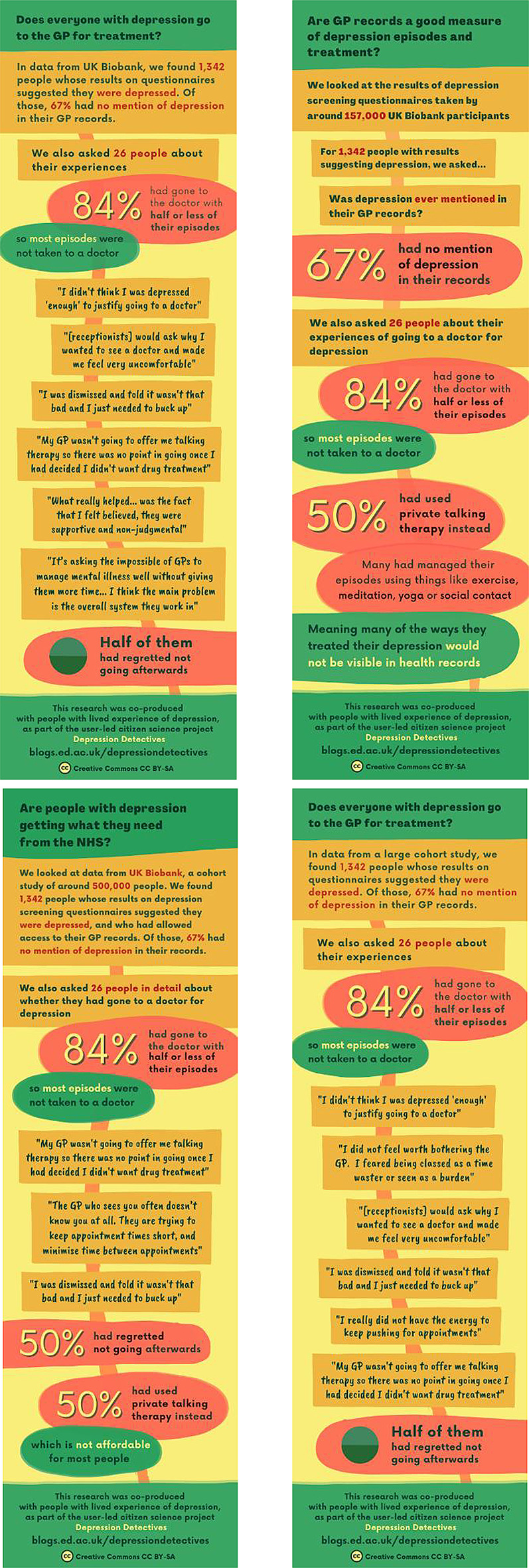
Infographics for GPs, Researchers, Policymakers and Patients, which communicate the key messages the group wished to disseminate

Following discussion in the Facebook group, the key insights that the group wanted to disseminate were transformed into infographics (see Fig. 9).

These results provide crucial context to the data science findings, enabling a deeper understanding of both the causes and the detailed experiences of patients. This insight is central to the project’s purpose, particularly for participants with lived experience.

### Project evaluation

#### Participant feedback survey responses

The survey was completed by 11 females, 2 males and 1 person who did not answer this question. Most (10/14) did not work in a mental-health role, but felt comfortable (7/14) or very comfortable (5/14) reading research papers or articles/books that describe scientific research.

People’s motivations for getting involved in the project fell into one of four main themes.

- to learn.
- to increase their own agency.
- to contribute to research.
- to help others.

When asked whether the project met their expectations, most were very positive. People found it informative, memorable, and engaging:

- “The enormous wealth of knowledge on depression helped me understand the various manifestations of the illness.”
- “[Researchers using their own lived experience] worked really well and helped sustain interest.”
- “I found myself getting more and more excited about participating. Feeling included and contributing to a quite unique project.”
- “It was quite an emotional journey at the beginning of the project.”

However, some found it overwhelming and hard to keep up with.

- “I found it difficult to keep up with all the posts on Facebook. This was probably more to do with my own workload and low energy levels than the way the project was run.”
- “Mostly I felt rushed and didn’t have the time to do things on your time scale”.

Talking directly to researchers was important for increasing knowledge.

- “The childhood trauma Q&A was really insightful. If I had not heard from those researchers, I might never have known stuff!”

A key impact of the project on participants was increased understanding of depression, in all its diversity:

- “I appreciate that labels like ‘depression’ can cover a huge diversity of experiences, that there is no scientific golden bullet for managing depression, that we’re still figuring stuff out individually, scientifically, as a health community.”
- “I realised the diversity of experiences with depression and the lack of a one-size-fits-all solution.”

Other impacts included opportunities for reflection and for reducing judgment.

- “It was really interesting to be able to take some time to really think deeply about my experience of depression and the journey I have been on. I’m not sure if I realised how much work I had had to do alone and with no guidance or support, but also how far I had come and what an incredible achievement it is to finally feel like I have come out the other side and survived.”
- “I think I am less judgmental of myself and others.”

Some felt they had gained the skills and confidence to pursue further independent study into depression.

- “I feel better equipped and more confident to question things.”
- “I intend to [explore the subject further] when I have more time”.

Overall, it was a positive experience that increased interest and knowledge.

- “My mental health improved enormously through participating.”
- “It’s been a really interesting project - thank you so much for being brave enough to think out of the box”.
- “I have enjoyed the experience as it enhanced my knowledge of MH [mental health] research.”

Some participants wanted more interaction with researchers and insight into their daily work.

- “I would like to have got more of a view into the day job of researchers to see how they were analysing the data and how they tried to answer our questions.”

#### Summary of participant interview findings

The interviewer conducted semi-structured interviews with four participants via Teams, lasting about 45 min each. Communication issues arose due to poor internet connections for two participants. Transcriptions were anonymised and analysed for themes, with names replaced with pseudonyms: Suzanne, Frank, Sally, and Jack.

#### Sample and participation spectrum

This small sample isn’t generalizable, but it revealed a spectrum of readiness for research involvement. Jack was highly motivated and engaged, while Suzanne would have preferred more guidance and structure. Frank and Sally fell in between.

#### Motivation for participation

Participants joined Depression Detectives to broaden their knowledge about the research process, to manage their depression better, and to contribute or give back to others. All enjoyed the project but found some aspects challenging.

#### Online platform

Facebook was considered an effective platform for reaching people while allowing a degree of anonymity. Jack noted it was particularly successful at this point in time, as the COVID-19 pandemic increased people’s willingness to connect online. “I’m in lots of groups, and in other groups the interest usually just fades; it’s hard to sustain it. I really think it worked in this group.” (Jack).

Concerns were raised about privacy and data security, although Sally felt these issues would have been more pronounced on the Zoom platform. Some participants expressed reservations about the overlap between the project and their personal lives, and all interviewees mentioned the need to take screen breaks for their mental health. Sally suggested using additional channels, such as WhatsApp, for important reminders.

#### Active participation experience

Participants held varied views on the co-produced nature of the project. Jack praised the democratic, user-led approach and found it educational, enhancing both his motivation and critical perspective on different types of research and their limitations. “I didn’t know before how to design a study and all the different factors you have to think about… and the analysis – I had not done this before, […] and it was a great opportunity to find out more.” (Jack).

Sally felt increased agency by having a role in setting the agenda.

Suzanne wanted to contribute her lived experiences but was less interested in conducting research herself, often referring to it as “your research” rather than “our research.” She expressed a preference for clearer guidance from researchers and felt uncertain about the project’s direction. “Being user-led means that there are too many voices and no clear direction”. Despite her reservations, Suzanne emphasised that she had gained a better understanding of research processes, including ethics applications.

#### Outcomes

All interviewed participants expressed uncertainty about the usefulness of the research outcomes. For them, “usefulness” meant making a difference for people with depression. Suzanne was particularly explicit: “There wasn’t a very definite outcome… this was a bit of - not a let-down, but kind of, uhh: right, what do we do with this now?… I don’t know how the research you did will help me.” (Suzanne).

However, the project clearly impacted them personally. They realised depression’s complexity. Suzanne noted her episodes varied in length, context, and cause, complicating treatment and research.

Jack also highlighted the complexity: “What was really interesting… the sort of complexity in people’s lives that might have contributed to depression… It’s not just depression, it’s two or three things at the same time…like autism or ADHD… Being in the group widened [my understanding] as people were bringing more associations and comorbidities.” (Jack).

Sally reported becoming more sceptical of generic questionnaires and RCTs (Randomised Control Trials), advocating for holistic, individualised and long-term research methods to capture depression properly.

“We really need to look at these things long-term and more holistically… If the goal of research is to have a healthy population, we’re not asking the right questions in the right timeframe… Depression Detectives helped to crystallise these thoughts.” (Sally).

#### Learning processes regarding participants’ own mental health

A significant amount of implicit learning occurred. Jack now plans to approach healthcare providers differently: “I have diabetes, and now I would always mention this to the doctor, which I didn’t do before”, “I would want a bit more time to think about different treatment options.” (Jack).

Suzanne, although often critical of the co-production of research, enjoyed the project and appreciated the opportunity to share her opinions. It prompted her to reflect on her mental health history:

It was thought-provoking… I realised I had several episodes of poor mental health. Actually, this wasn’t just a single episode. This is something that has gone on earlier in my life as well. ….I wouldn’t have sat back and examined my earlier years in such a way normally. (Suzanne)

For Suzanne and others, the project had a quasi-therapeutic effect, offering a space to reflect more deeply on their experiences with depression and to compare these with others, helping them to construct new narratives around their mental health.

#### The value of sharing among peers

Participants consistently highlighted the value of sharing personal stories and hearing others’ experiences. “I really liked the peer exchange and hearing what others had been through… There was a therapeutic aspect of telling one’s story - it puts it in the past… it was a privilege to hear other people’s experiences.” (Frank)

It was good to have a place to talk about my own experience… Seeing similarities with others… Many would feel better if they just felt heard or recognised. It’s not happening through the NHS… So how do we do it for each other? (Sally)

Jack noted that hearing researchers share their own lived experiences helped foster openness and trust:

That actual researchers were sharing their lived experience made me feel well understood. I think it can create an environment where people probably shared a little more and with a little more comfort… it felt like a really open way to do things. (Jack)

Although Depression Detectives was not designed as a peer support initiative, participants greatly benefited from the opportunity to share and listen in a supportive environment.

#### Future projects

All interviewees expressed interest in participating in future projects such as Depression Detectives and indicated they would recommend it to others. They suggested focusing on specific themes such as depression and menopause, the impact of bullying, or underrepresented groups, to deepen engagement and relevance.

#### Researcher feedback survey responses

Eight researchers completed the survey. Seven found the project thought-provoking, four called it good, while three described it as new, fun, and frantic. Two found it stressful. None described it as easy or exhilarating. In terms of confidence with public engagement, five felt more confident, and the others felt about the same.

Some researchers found the Q&As stressful, worrying about saying the wrong thing:

“The Q&A sessions were very frantic, and I worried about posting something in the spur of the moment, when normally putting something out in the public domain, I would try and be very careful/considered.“

I found the Q&A format quite stressful.

Not feeling like I was qualified enough to give an opinion. ….I was anxious about saying the wrong thing, being misleading or sharing false information.

“At some point, the questions were unstructured and, to some extent, overwhelming.”

The researchers also highlighted several benefits, including understanding the views of those with lived experience: “I enjoyed hearing the public’s thoughts on our research.”

Answering questions in the Q&A and finding out the interests of participants.” “Really useful to get the opinions of people with lived experience over time - better than just having one or two meetings.

They also gained new insights: “Some interesting perspectives on the topic that I hadn’t previously considered”, and improved their communication skills, " to learn how to use lay language to communicate research findings.”

In terms of explicit learning, the researchers mainly spoke about their insights into public perspectives:

It reminded me that the public don’t have a lot of detailed knowledge about how research works….might need to be explained.

How interfacing with healthcare, especially waiting for psychological treatment, contributes to the experience of depression.

I realised that there are a lot more practical questions about how to treat depression rather than what may cause depression. I think it is sensible to consider bringing more translational aspects to our research.

#### Researcher interviews analysis

Five researchers were interviewed on Zoom by an independent researcher. The interviews were recorded, transcribed, and analysed by themes.

Interviewees held mixed views on the use of Facebook. While it was seen as an effective communication medium, appreciated for its familiarity and closed-group option, concerns included distrust of Meta (Facebook’s parent company) regarding ethics, privacy, and its age demographic.

Researchers valued interactions with the participants, finding them insightful and beneficial for gaining a deeper understanding of different perspectives. They appreciated the two-way discussions and that both they and the participants could ask follow-up questions.

Two out of five researchers expanded their research ideas, and two found the results more interesting than expected. One considered new research areas that could be explored, while another was surprised that the project had produced publishable results, surpassing their expectations.

The Q&As were again described as intense, with the additional stress or anxiety about answering in written form, which other researchers could later read on a website.

All interviewees wanted the project to continue or be repeated, suggesting a longer duration to build trust. They felt the current project was too short.

Researchers cited their introverted nature as a barrier to public engagement. Furthermore, they felt that engaging the public with this complex field of research presented additional challenges due to the sensitive nature of the health data involved. Views ranged from believing public involvement in the ‘tricky bits’ (in-depth data analysis) was impossible, to suggesting that methods could be developed to make it possible and understandable.

The interviewer felt that there was often a misunderstanding of the term ‘co-production’ as she carried out the researcher interviews. 2 out of 5 researchers interviewed believed lived experience members should only help with dissemination, and not be involved in the data analysis stage at all, due to the sensitivity and potential identifiable nature of the data. This reflected their differing views on public involvement in research.

## Discussion

The study used a co-production approach, in which lived-experience participants played an active role in shaping and conducting the research, from selecting the topic to disseminating the results. This exemplifies respect for epistemic justice by valuing participants’ experiential knowledge alongside academic expertise, as advocated by Smith et al. (2021)[5]. Many of these participants found unexpected benefits through peer support, which helped deepen their understanding of depression. The research project itself highlighted a big gap in how often depression is recorded in GP records compared to other health issues. Participants expressed mixed feelings about the term “episodes”, with some feeling their condition was more chronic. The participants also described barriers to seeking help, such as feeling they weren’t “depressed enough” or having had negative experiences with GPs in the past, who seemed rushed and under-resourced. There was a strong desire for more holistic research and for more treatment options to be available via the NHS.

There are other ways to get insight into the lived experience of people who experience depression - e.g., mixed methods research using interviews, surveys, etc. but co-production is also about changing power dynamics. You don’t know what lived experience participants really care about until you provide the opportunity for them to decide, and give them some time and informational support to think in depth about the question. Our co-production methodology, which took place over several months, enabled both groups (lived-experience participants and researchers) to develop a shared understanding of one another and of their respective perspectives. People could go away and reflect on what they’d learned and come back with more thoughts or understanding. Lived experience participants often mentioned how much they learned from their interactions with other participants, and the context that gave to their own experiences of depression.

### Question selection process

We used a two-stage process to choose the research question for the group to focus on. This was a strategic decision designed to enhance participant engagement and satisfaction. In previous projects, such as Nappy Science Gang [47], a single-stage process saw participants vote for broad questions. Which then had to be narrowed down to design a practicable experiment, resulting in disappointment for some participants. Although this provides a valuable insight into the research process, it can be disappointing for participants and lead to disengagement (*“If I had understood that we would only be answering PART of question A*,* then I would have chosen question B”*).

The two-stage process we used here, first developed during the Parenting Science Gang [39], had the group vote on a long list to choose a top ten. Then, explore those top ten questions in detail before making a final choice. This provides a space for communication about the complexity of research. The daily ‘spotlight’ posts enabled collaboration with researchers and facilitated communication regarding budget, time, and practical constraints. By ensuring participants understood the research limitations, we aimed to avoid post-vote regret.

As researchers and public engagement practitioners, our job is to design processes that provide equitable access to information, enabling public participants to make informed decisions.

Furthermore, one of our core principles is allowing participants to choose their level of involvement. This two-stage process, with multiple entry points for asking clarifying questions or adding comments, facilitated this flexibility.

### Ethics and participation

Ethical considerations presented significant challenges, mainly due to the absence of clear procedures for obtaining ethical clearance for public engagement/PPI projects. However, these challenges are widespread across the sector, as noted by The Co-production Futures Inquiry [51] and in consultations by the NCCPE [52] and MRC [53].

The Edinburgh Medical Research Ethics Committee (EMREC), which accepted our application, though supportive and accommodating, was not set up to consider co-produced research projects in which the study participants were also the experimenters. Their approach was inherently conservative and protective of our ‘vulnerable’ participants. Their processes were also poorly suited to a time-bound project in which the question and method were unknown at the outset. Having to return to the ethics board partway through introduced delays and methodological restrictions, which our participants and researchers found frustrating.

The presumption of vulnerability with regard to depression raises questions about autonomy and capacity – especially when estimates suggest up to 25% of women and 12% of men will develop depression in their lifetime [54] - and indeed 50% of the population is likely to develop a mental disorder of some description by the time they are 75 [55]. Should they all be treated as vulnerable and protected from their own decisions in perpetuity?

Scottish law presumes that adults have the capacity to make decisions [56]. The starting point is a presumption of capacity, which can be overturned only where medical evidence demonstrates otherwise. Furthermore, the Adults with Incapacity (Scotland) Act 2000 [57] establishes in law the principle of presuming capacity as far as possible, respecting people’s choices, and using the least restrictive way to achieve the purpose. The lengthy consent documents used in this study seemed misaligned with its public engagement goals. (See Additional Information 3, description of process in ‘Ethical Considerations’ section of methods and more information in Table 1). A possible solution is a two-step approach, in which participants first join an initial group to better understand the required level of involvement before committing to the entire process. This method would better align with the project’s spirit. However, any adjustments to privacy protocols must be carefully considered from an ethical standpoint and may be more feasible on a platform where participants’ identities are concealed.

Only people who were not currently experiencing depression were supposed to sign up to participate. However, given how many of our participants described their depression as chronic, with one episode merging into the next, it is unclear if this was a realistic request. Is it always easy to be sure one isn’t currently depressed? Several participants talked about realising in retrospect that they’d had periods of depression which they hadn’t recognised at the time. Further, some members described it as infantilising or patronising that they weren’t trusted to make their own decision about whether it was safe for them to participate.

The University of Edinburgh’s intention to establish a specialised PPI ethics group [Personal Communication with IB] is a promising development in addressing such challenges, and it is encouraging to see that other universities and organisations are taking similar actions [51].

In general, we advocate for a system in which PPI ethics and safeguarding procedures are assessed by PPI experts, who have the skills and experience to evaluate the expected levels of diversity and vulnerability within the study population. Achieving a balance between adequate safeguarding (which fosters participation) and overly complex procedures is challenging. Therefore, further discussions, research, and consultations with patients and the public around this topic are essential.

### Limitations of data science methods

The UK Biobank is a valuable resource for studying how various factors relate to disease, owing to its large and detailed dataset. However, it’s important to note that participants are generally healthier and wealthier than the average UK population [58–60]. Additionally, although the Biobank includes a variety of socioeconomic backgrounds, it has limited ethnic diversity, reflecting UK demographics at the time the data were collected [58]. These factors can affect the strength of some findings (the exact numbers reported in the study). However, they do not usually affect the presence or absence of the relationships themselves (in this case, self-reported vs. GP-recorded depression diagnosis), which can still be meaningfully applied to the broader population [60].

It should also be noted that we made use of self-reported depression in both quantitative (data science) and qualitative (survey and focus group) aspects of this study. Self-reported depression helps researchers capture a broad range of symptoms and experiences, which enriches our understanding of depression’s complexity [61]. Genetic correlations highlight the effectiveness of self-reported depression in identifying genuine risk factors [62]. The scalability of self-reporting also makes it practical for both PPI and large-scale research studies, yielding valuable insights that complement clinical assessments [59, 63].

Using GP codes to measure depression also has limitations. The 67% of cases without a recorded diagnosis could be due to patients not disclosing symptoms or to GPs documenting mood-related symptoms in the written notes without confirming a depression diagnosis with a diagnostic code [64]. GPs also code conditions like PTSD (that have mood symptoms) with a separate code, so the patient may report depression, but the GP has coded PTSD. All of these (and potentially other things) could result in a lack of depression ‘read codes’ in patient notes, which would affect our numerical results (i.e. the 67% figure). However, as reported above, the relationship between the two factors (self-report and GP records) is unlikely to disappear.

### Project dynamics: chaotic or fun?

In general, participants were very positive about the project’s user-led nature. They found it engaging and appreciated that their voices mattered. However, in the interviews, participant ‘Suzanne’ felt quite unsettled because she didn’t know where the project was going. Whereas another felt exhilarated and excited by it. This wasn’t an angle we had asked evaluation survey questions about, so we don’t know how many other participants felt the same way.

The researchers, by contrast, often reported that Q&A sessions were stressful and overwhelming. In a way, they were all Suzannes. In previous projects using online Q&As (e.g. Parenting Science Gang [39], or the earlier I’m a Scientist, Get me Out of Here [65]), the hecticness was described as ‘buzzing’, ‘exhilarating’ and ‘fun’ by researchers. In the current project, researchers used words like ‘stressful’, ‘anxious’ and ‘difficult’.

We don’t know whether these differences in how people felt in response to uncertainty and light chaos reflect individual personality differences, differing expectations, or another factor, such as the sensitivity of the research topic or fear of release of views into the public domain. It is notable that in both Parenting Science Gang and I’m a Scientist, Get me out of Here, the researchers had individually volunteered themselves. Whereas in Depression Detectives, the department had signed up for the activity, and individual researchers had less agency about their involvement.

It will be interesting to reflect on and perhaps explore these dynamics as we design future projects. Can we predict who might find it challenging or rewarding, and can we support them to frame uncertainty positively? With major funders pressing for more co-produced research [66], addressing these questions is crucial.

### Reflecting on the model

We have now used several iterations of this model (Nappy Science Gang [47], Parenting Science Gang [39], and Depression Detectives), while continuously developing and adapting it.

An essential part of the model is the space it creates, where lived experience participants are in the majority. This allows them to steer the conversation and discuss whatever topics they wish. This results in more in-depth, wide-ranging discussions, uncovering unexpected topics and exploring seemingly unpromising paths to uncover valuable insights.

Continuous conversation and interaction also create useful feedback loops. By analysing popular content and gathering participant insights and requests, the project team can refine their approach and ensure the content remains responsive to the group’s needs and promotes inclusivity. For example, if the group are asking lots of questions about a particular topic, the facilitator might find and add relevant copy, arrange a Q&A on this topic, or invite a specific researcher to join the group to aid with these discussions.

Our setup also fosters a social environment that emphasises building connections over simply progressing through an agenda. Participants experience genuine solidarity and fellowship, reducing feelings of isolation. This is especially crucial for topics like depression and early motherhood, but it also applies to many other health conditions that can be isolating. We believe that this format, or similar ones, can effectively support co-production across a wide range of subjects.

A crucial aspect of this model is that participants can adjust their level of involvement. Participating and pausing as they wish or as life permits. This allows for the inclusion of a larger and more diverse group of people than would be possible in a formal, fixed 'PPI committee member' role, for example.

We used Facebook for its convenience, familiarity to our intended participants and broad reach, while recognising its limitations. A similar project could employ a different platform suited to the audience, as long as it supports threaded conversations for asynchronous interactions and offers mass appeal with low barriers to entry.

Common feedback highlighted difficulties in selecting questions that are universally appealing. Within the Parenting Science Gang [39], groups with a narrower focus found it easier to choose a question and retained more active group members over the longer term. Participants in this Depression Detectives project also suggested that it would have been interesting to have specific subgroups, e.g., depression and autism, depression and menopause. A future project that incorporates a range of special-interest subgroups could yield highly valuable results.

Feedback from both participants and researchers highlighted feelings of being rushed and of being surprised by time and logistical constraints. Partly, this may be because the lived-experience participants were not familiar with the research process. For the researchers, it was a very different experience from their usual research. Some found transitions between project phases abrupt and often felt uncertain about progress or next steps. We received similar feedback on previous projects [39, 47] and believed that this time we had been much clearer. We provided scaffolding, repeated messages, and simple timelines, but we recognise the need to further enhance these in future projects. One participant interviewee suggested a separate communication channel for key milestone reminders, such as text messages, WhatsApp, or email. We believe this “mixed methods” approach is worth exploring. We also acknowledge that this pilot was very short (8 months) and that a longer project (perhaps around 2 years, as was the case for Parenting Science Gang [39]) would provide more time to work effectively through all of the stages.

One way to support both researchers and participants would be to offer training. This could cover topics such as co-production principles, effective communication and research methods, as well as ‘how to’ guides, if the platform itself was less familiar. For researchers specifically, concise videos and infographics that explain the co-production concept, their role in the project, and the expected time commitments could be very useful. Overall, training would help equip all participants with the necessary skills and confidence to engage fully and meaningfully and further improve the model’s inclusivity.

Additionally, some of the researchers felt nervous about saying the wrong thing during the Q&A sessions. Providing the researchers with an “add notes” option before publishing the Q&A transcripts on the blog could increase their confidence that inadvertent errors could be corrected.

Several participants emphasised the importance of effectively communicating findings to influence policy and practice. However, academic or clinical research often struggles to achieve this kind of impact [67]. In future projects, we would aim to describe these challenges and potential solutions more clearly to participants. Although we did broadly discuss with the group what they wanted to achieve with our research and what they would change about depression care (e.g., during the question selection process and in one of the Q&A sessions), we observed a general decline in participation during discussions about dissemination. If given a longer project timeline and additional resources, we would have included more Q&As and earlier discussions on dissemination. This approach would have helped maintain a focus on outcomes and target audiences and allowed participants to consider how each project decision affected these goals.

In future projects, we would also seek to co-produce the evaluation as much as possible. Some evaluation work is necessarily done before participants are recruited (e.g. writing baselining ‘pre-event’ surveys for participants). But participants can be partners in deciding what you ask later, and how, and what it’s important to measure. They can also help design pre-event surveys for future co-production projects, as proxies for future participants. We would also gather additional evaluation data, including more concrete measures of engagement, such as popular activities and individual participation rates throughout the project. It is well established that there is a steep participation inequality in online communities, with a small number of users contributing most of the content [68, 69]. In short, some people prefer to lurk, observe, and perhaps vote or respond to polls. Others feel highly committed or enjoy posting and generating content. It should also be noted that people may move in and out of active participation over time for reasons that are partly project-related (e.g., whether they find this part of the process interesting) and partly not (e.g., a new job, a new baby, illness). The design of this project specifically allows people to dip in and out and contribute at levels that suit them.

### Impact of the project

The time and resources invested in this user-led citizen science project have delivered both scientific and societal value. Engaging participants directly revealed insights that traditional methods may have missed, most notably that most depressive episodes are not taken to a doctor. For data scientists, this highlights that electronic health records and prescription data alone provide an incomplete picture of depression, as many self-directed coping strategies remain undocumented. For clinicians, the findings clarify barriers to seeking help, from perceptions of symptom severity to preferences for counselling over medication, with participants suggesting practical improvements such as automated telephone triage options for mental health. At the policy level, the project highlighted the need for longer consultations, continuity of care, and service diversification beyond drugs.

Within our research group at the University of Edinburgh, the project prompted discussions on episodic versus chronic depression, influenced a PhD thesis, and informed grant applications [31]. Dissemination of this project has reached local and international audiences through, for example, scientific and PPI conferences [70–75], shortlisting for The Nature Awards for Inclusive Health Research [76] and inclusion in their Best Practice toolkit [77], university wide articles (University of Edinburgh), and contributions from Depression Detectives members to the £4.5 million Wellcome Trust–funded AMBER project [31], to shape the participatory elements and knowledge exchange activities.

Overall, this project demonstrates that user-led citizen science produces substantial return on investment. Embedding lived experience uncovers insights that would otherwise remain hidden, strengthens applicability to practice and policy, and empowers participants, fostering meaningful impact across multiple stakeholder groups while promoting a culture of co-produced, patient-centred research.

### Considerations

Finally, we want to emphasise how time-consuming and staff-intensive a co-produced project like this can be. As participant ‘Jack’ said, when interviewed, “I’m in lots of groups, and in other groups the interest usually just fades; it’s hard to sustain it.”

We had four members of engagement staff spending one day a week on the project, posting, reminding, finding interesting content to share and prompting discussion. We also had 30 researchers from the department available to answer questions and participate in Q&As. We held regular ‘events’ to keep interest high and, of course, had the lure of conducting research together.

The relational and administrative demands are significant, particularly when they occur within systems that are not designed for such work [51]. Authentic collaboration with researchers, ample time, and an enthusiastic, supportive team are essential for success.

## Conclusion

The Depression Detectives project successfully demonstrated a method for bringing together people with lived experience and university researchers to co-produce research over an extended period, in ways that were accessible to a wide range of participants and had low barriers to entry.

The research that the group co-produced highlighted the underreporting of depression in GP records and the complex barriers individuals face when seeking medical help. Our findings underscore the need for more holistic and accessible treatment options within healthcare systems.

Participants valued the opportunity for peer support and the chance to directly influence research, finding the process informative, engaging, and transformative. Researchers gained valuable insights into patient experiences, although they found the process challenging at times.

Moving forward, there is a clear need to address ethical and logistical challenges to better support participants and researchers. As co-production becomes more prominent, understanding its dynamics will be crucial for future projects to ensure they are both impactful and inclusive.

## Electronic Supplementary Material

Below is the link to the electronic supplementary material.

Supplementary Material 1: Initial Sign-up form Includes Patient information sheet, eligibility check and questions about personal contact details, emergency contact details and evaluation questions

Supplementary Material 2: The top ten research questions, as voted for by the Depression Detectives participants

Supplementary Material 3: Co-produced Survey Questions and results. Includes Patient information sheet, survey questions asked and results as graphs & copies of the open text answers

Supplementary Material 4: Evaluation Form Questions Includes the questions presented to participants and researchers about their experiences of the project

Supplementary Material 5

## Data Availability

The top ten research questions, co-produced survey questions and results, initial sign-up form and evaluation form questions have been included as additional information. The other datasets used and/or analysed during the current study are available from the corresponding author on reasonable request.

## Abbreviations

AMBER: Antidepressant Medication: Biology Exposure and Response
ADHD: Attention Deficit Hyperactivity Disorder
CBT: Cognitive Behavioural Therapy
CIDI: Composite International Diagnostic Interview
CIDI-SF: Composite International Diagnostic Interview – Short Form
DD: Depression Detectives
EMREC: Edinburgh Medical School Ethics Committee
GP: General Practitioner
Jisc: Joint Information Systems Committee
MRC: Medical Research Council
MQ: The name of a Mental Health charity in UK
NCCPE: National Co-ordinating Centre for Public Engagement
NHS: National Health Service
NIHR: National Institute for Health and Care Research
PHQ9: Patient Health Questionnaire 9
PPI: Patient and Public Involvement
Q&A: Question and Answer
RCT: Randomised control trial
REF: Research Excellence Framework
SiP: Science is People Ltd
UK: United Kingdom
UKRI: UK Reearch and Innovation
UNESCO: The United Nations Educational, Scientific and Cultural Organization
