# Supplementary material for: Depression detectives: piloting a methodology for online co-produced research
Source: Res Involv Engagem. 2026 May 18;12:62. doi: 10.1186/s40900-026-00889-2 (PMC13182061; doi:10.1186/s40900-026-00889-2)
Supplement: Supplementary file 1 — Supplementary Material 1: Initial Sign-up form Includes Patient information sheet, eligibility check and questions about personal contact details, emergency contact details and evaluation questions [file 40900_2026_889_MOESM1_ESM.pdf]

Additional Information 1

Initial Sign-up form

Includes Patient information sheet, eligibility check and questions about personal contact details, emergency contact details and evaluation questions.

Depression Science Gang - Initial sign up Form

0%

0% complete

Page 1: Page 1

Sorry for the length of this information sheet and the form that follows. We want to make sure you have all the information you might need, before you decide whether to join the group.

Participant Information Sheet

You are being invited to take part in Depression Science Gang – a user-led citizen science project about depression research.

Citizen science projects invite the public to get involved in some way.
User-led refers to allowing volunteers to set the agenda; they will choose the questions that the project will explore and co-design/run the experiment alongside the researchers. (And hopefully also learn some stuff and meet some interesting people along the way).
Each volunteer gives as much or as little time as suits them.

Read more about a previous user-led citizen science project called Parenting Science Gang.

Dr Iona Beange, Knowledge Exchange and Impact Officer at the University of Edinburgh is leading this project.

Before you decide to take part it is important you understand why the project is being done and what it will involve. Please take time to read the following information carefully.

What is the point of this project?

The purpose of this project is to give people with lived experience of depression a bigger voice in research. You know things, and have insights, that researchers may not have. We think research will be better and more useful if we bring together people with lived experience, and scientists, to discuss things together. The first thing we will be looking at is: what do people with lived experience of depression think is important to research?

Why have I been INVITED to take part?

You are invited to participate in this study because you either have lived experience of depression.

Do I have to take part?

No – it is entirely up to you.

If you decide to take part, please keep this Information Sheet and complete the Informed Consent Form to show that you understand your rights in relation to the project, and that you are happy to participate.

What will happen if I decide to take part?

You will be sent a link to a private Facebook group, where we will host our discussions. This group will NOT appear on your own or your friends' newsfeeds.

Within the private Facebook group we will:

- Host discussions to seek your opinions/ views/ inputs
- Run polls
- Bring researchers along to talk to you and answer your questions.
- Choose questions that you would like scientific answers to, and hopefully design a small citizen science project that researchers and volunteers can carry out together (we will take you through the process step-by-step).
- We may also run some practical online sessions to explore treatment options (e.g., mindfulness), depending on the group's interest.

The Q&A sessions will be written-up for the project blog, but all names and personal identifying details will be removed before we make anything public..

We may also summarise and write about some of the other discussions that take place in the private Facebook group (e.g. progress towards a list of questions / areas that we are interested in), but again, contributions will be anonymised and summarised before being reported.. No names or identifying details will be made available outside of the private group.

This pilot project will last until July 2021.

(We will try to get money for a longer version of the project, but it will be up to you whether you want to take part in the second project or not).

What are the POSSIBLE benefits of taking part?

By sharing your experiences and opinions with us, you will be helping to make depression science better, and more informed by what people who've experienced depression want and know.

We also hope that you will enjoy it and get a lot out of it.

In previous projects a bit similar to this (but not about depression), people who took part said that they learned a lot, and felt more confident talking about science, making decisions about science, and talking to healthcare staff.

Similarly, talking to others and having a chance to directly speak to researchers/ask questions can be rewarding.

Are there any risks associated with taking part?

There are no significant risks associated with participation.

However, by taking part you may be revealing your history of depression to the project staff and to the other members of the private group. (NB: any reports/blog posts etc that are made available on the website will be anonymised).

Taking part could bring back distressing memories or trigger depressing thoughts.
We ask that you do not take part in this project if you are currently in a state of crisis and that you remain aware of your own mental well-being throughout. It is fine to take breaks from the project when you need to do so, and you are welcome to come back in when you are ready.

We would also encourage you to create your own 'Wellbeing and Safety Plan' (download it here).This is a document which you can hand to a friend or relative, which describe your personal 'warning signs' and what you would like to happen if these are observed (e.g. talk to me about how I'm feeling / encourage me to call my doctor). You may wish to share these warning signs with our project staff, but you don't need to do this.

A list of helplines and sources of information are included in the Wellbeing and Safety Plan and will be provided as a pinned post in the Facebook group.

Our staff will do everything they can to help you with the project, but please note that they are not able to offer mental health support and will not be available at all times.

During the sign-up process we will ask you for a telephone number, so that we can contact you should we become concerned about your wellbeing. There will also be an option to

nominate a friend or relative that we could contact to support you if required (providing these details is optional and we would always aim to discuss options with you first before contacting anyone else.)

WHAT IF I WANT TO WITHDRAW FROM THE PROJECT?

Even after completing the consent form, you are still free to withdraw at any time and without giving a reason. Please just tell Iona Beange (iona.beange@ed.ac.uk) so that we know not to contact you anymore.
Or if you decide to take a short break from the project – that is fine too.
All participation is optional and you can come back in whenever you are ready.

If you withdraw from the project, we will ask if we can still make use of the comments/opinions that you have made so far, or if you want us to delete them. That decision is up to you.

However, you should note that if your views/opinions have already been used in the production of formal project outputs (e.g. blog posts, project reports, journal articles, conference papers) prior to your withdrawal, it may not be possible to remove these.

Similarly, we may need to keep a record of your name and period of participation for our record keeping (all other personal data will be deleted).

Taking part in the pilot project does not oblige you to take part in any follow on projects. All participation is optional.

Data Protection

Your data will be processed in accordance with Data Protection Law. All information collected about you will be kept strictly confidential.

The personal information you provide on the initial sign-up form will only be accessible by Dr Iona Beange and Professor Andrew McIntosh at University of Edinburgh.

- The answers to the initial questions will be used to check your eligibility for the project.
- The email address will be used to provide you with the link to the private Facebook Group and to send you project updates (if you agree to that).
- The personal details / emergency contact will only be used if we become concerned about you or those around you. It will be deleted at the end of the project.
- The demographic information will be summarised for evaluation purposes.

The initial sign up forms will be deleted at the end of the pilot project. Only summary evaluation data will be retained.

Staff at Science is People Ltd will be able to see your responses on the private Facebook group and may receive your anonymous evaluation data for analysis (after it has been checked for any accidental disclosures by Iona Beange, University of Edinburgh).

All forms will be stored within password-protected computer systems.

Any reports, summaries or blogs that are made available to the public will always be anonymised (unless you explicitly ask us otherwise – e.g. if you write a blog post for us, you may wish to put your name on it).

Anonymous evaluation data may be retained after the end of the project, but all personal information will be deleted. The Facebook group itself will be deleted with 6 months of the end of the project.

A key output from this process will be a list of questions that will be useful to researchers when making decisions about what to study in future. This list will be stored for several years, but will not include any personal data.

Confidentiality and reporting obligations

Any information that you disclose during the project will be kept confidential, unless our staff feel there is a direct risk to your own safety or the safety of others. At this point it may become necessary for our staff to inform the relevant authorities.

Specific reporting obligations exist for child protection offences (physical or sexual abuse of minors), the physical abuse of vulnerable adults, money laundering and other crimes covered by prevention of terrorism legislation. If you reveal any of these things, our staff will be obligated to report these to the police or relevant authorities.

Posts that encourage others to take part in illegal activities will be deleted.

International Data Transfers

For this project we will be making use of Facebook. Under the terms of GDPR, that puts a joint responsibility for the data onto both us (University of Edinburgh and Science is People Ltd) and Facebook.

Their official address is Facebook Ireland Ltd. (hereinafter "Facebook"),
4 Grand Canal Square Grand Canal Harbour Dublin 2 Ireland

Depending on your privacy settings, Facebook will have access to a certain amount of personal data about you.

You may find the following links useful:

- Facebook's data policy: https://www.facebook.com/policy.php
- Facebook's education module on privacy and how to change your settings: https://www.facebook.com/about/basics
- Facebook Inc., the US parent company of Facebook Ireland Ltd., is certified under the EU-U.S. Privacy Shield and thus promises to comply with European data protection guidelines.

The University of Edinburgh and Science is People Ltd may access some of this data via Facebook analytics ('Group Insights') and use them for evaluation purposes. However, even if we do not access them, we have no control over the data that Facebook collects and processes in the background.

What will happen with the 'results' of this project?

The main piece of data ('results') that we are collecting are your interests/opinions/questions in relation to depression research. These are expected to come out during the discussions and Q&A sessions within the Facebook group.

The Q&A sessions will be anonymised and reported on the project website/blog.
If we decide to write up any of the other discussions, they will be summarised and anonymised before they are reported outside of the private group (e.g. in blog/website posts, published articles, reports or presentations).

Your name will only be used in very limited situations where we have your prior and explicit written permission (e.g. you wrote the blog post and want us to put your name on it as author).

Discussions within the group and responses to the evaluation forms may also be used for evaluation purposes and to inform/improve future projects. This anonymous and summarised data may be retained after the end of the project.

There may also be 'results' generated by the pilot citizen science project. These may be reported on the project blog/website, in published articles, reports or presentations. You will be asked at the time if you want your name to be included (or not) in any publications etc.

WHO CAN I CONTACT?

If you have any further questions about the study, please contact the project lead, Iona Beange, Knowledge Exchange and Impact Officer, University of Edinburgh
– iona.beange@ed.ac.uk

If you wish to make a complaint about the study, please contact:

Hazel Lambert, Public Engagement with Research Manager, University of Edinburgh. Email: hazel.lambert@ed.ac.uk

In your communication, please provide the project title and detail the nature of your complaint.

You can get this document in audio, large print and various computer formats if you ask us. Please contact us on iona.beange@ed.ac.uk and quote project title.

For general information about how we use your data go to:

https://www.ed.ac.uk/records-management/privacy-notice-research

The Samaritans (www.samaritans.org) are a national listening service. If you feel triggered by this form, please consider contacting them for support. Phone 116 123

I have read and understood the information sheet and consent to take part in the project. *Required*

- Yes
- No

I understand that this is a joint project between the University of Edinburgh and Science is People Ltd and that both organisations will be able to see the information that I post on the private Facebook group and any data that is collected by Facebook analytics.

- I accept this
- I do not accept this

I understand that this project is designed to collect ideas and opinions, so posts that I make in the private Facebook group / during Q&A sessions may be anonymised and summarised, then written into posts for the website/blog and reports

- I accept this
- I do not accept this

- Next

Page 2: Eligibility to take part

I am 18 years of age or older *Required*

- Yes
- No

Do you identify as having lived experience of depression? *Required*

- Yes
- No

For your own safety, we ask that you make an assessment of your own mental health at this point. If you are currently unwell or feel that this project may trigger your depression, we ask that you do not take part.

As noted on the information sheet, we also recommend that all participants make '[Wellbeing and Safety Plan]'. This is a document which you can hand to a friend or

relative, which describes your personal 'warning signs' and what you would like to happen if these are observed (e.g. talk to me about how I'm feeling / encourage me to call my doctor).

Are you currently experiencing a mental health crisis? *Required*

- ○ Yes, I am currently in crisis
- ○ No, I am not currently in crisis
- ○ Other

A Facebook account will be needed for participation in this project.

However, as signing up to Facebook comes with data protection implications, we do NOT recommend setting up an account because of this project. We are looking for existing Facebook users.

Do you have an internet connection and a Facebook account?

- ○ Yes
- ○ No

- Next

Page 3: Information so we can contact you

Preferred name (what do you want us to call you?)

Email address *Required*

How may we use your email address *Required*

- ☐ To send me the link to the private Facebook page (required)
- ☐ To send me project updates (optional)
- ☐ You may not use my email address for any purpose (this will exclude you from the project)

What is your name on Facebook (so that we know it is you and can give you access to the private group). *Required*

- Next

Page 4: Emergency Contact Information

If an emergency occurs and our staff believe there is a danger to yourself or someone . The only people that will be able to access this information are:

- Dr Iona Beange, Knowledge Exchange Officer and Project Lead
- Professor Andrew McIntosh, NHS Psychiatrist
- Dr Christine Kupfer,

By completing these details you are giving us permission to use them for the purposes described above.

Your official name (Which may be the same or different to the preferred name that was stated on the previous page). *Required*

Your telephone number *Required*

Name of friend, relative or someone else that you would like us to contact if we are concerned about your wellbeing. Who would be the most supportive to you?) *Optional*

Telephone number of emergency contact person *Optional*

- Next

Page 5: Evaluation

We believe that researchers do better research when they listen to people with lived experience. So a key aim of this project is to improve and inform research.

However, we are also interested in how the project affects you and how you feel about research/science.

Your answers in this section will only be taken into account after this project and will not influence our perception or you during this project.

Do you work in a mental health-related role or are you studying a mental health-related subject? (e.g. a researcher, counsellor, mental health nurse, work for a mental health charity or have some other mental health related role)?

- Yes
- No

Which of these statements best describes your attitude towards mental health research / the science around depression?

- I am against / distrust it / feel negatively towards it.
- I don't think about it at all
- I have some awareness of it, but I wouldn't look it up.
- I sometimes come across things informed by it, but I wouldn't look for them.
- I would like to know more about it, but the information is hard to find or understand.
- I sometimes look it up / read or watch things informed by it.
- I regularly look it up / read or watch things informed by it.
- I am very up-to-date with it and often read about it / articles informed by it.

Do you feel that research / scientific information is relevant to <u>your own personal</u> management of your depression?

- Definitely not,
- No,
- I'm not sure,
- perhaps,
- yes, definitely.

Do you have experience in engaging with research (on any topic)?
E.g. taken part in an experiment, studied it, attended a public lecture or science festival activity, I am a member of a cohort, work experience placement etc.

- Yes
- No

If yes, in what form? (text box)

How comfortable do you feel reading research papers or articles/books that describe scientific research?

5 4 3 2 1

Very comfortable intimidated.

Further thoughts on this question: (text box)

If you were not sure about something a healthcare provider says or have a different opinion, would you be able to ask or challenge them?

5 4 3 2 1

Very comfortable would stay silent.

Further thoughts on this question: (Text box)

- Finish
