# Supplementary material for: Depression detectives: piloting a methodology for online co-produced research
Source: Res Involv Engagem. 2026 May 18;12:62. doi: 10.1186/s40900-026-00889-2 (PMC13182061; doi:10.1186/s40900-026-00889-2)
Supplement: Supplementary file 2 — Supplementary Material 2: The top ten research questions, as voted for by the Depression Detectives participants [file 40900_2026_889_MOESM2_ESM.pdf]

Additional Information 2

The top ten research questions, as voted for by the Depression Detectives participants.

1. Do people with depression feel that they predominantly receive help to treat their “symptoms“ vs “origins”? How could this be changed?

2. What is the effectiveness of treatments on offer from GPs on the NHS (mainly anti-depressants and short-term counselling) and what proportion of patients recover with just this, what proportion go on to have a major crisis which enables them to access more in-depth treatment, and what proportion end up self-funding something which actually works in the long-term? [needs to be narrowed down]

3. How do people who say that they have recovered from depression describe their recovery: Do they think they are "cured" or just "coping better", "able to spot triggers better", etc.?

4. How does chronic depression/dysphoria differ from, say a single episode, or discrete episodes of reactive depression? Are there markers (biological, psychological, behavioural, and current or in a person's history e.g. trauma) that distinguish them? [narrow down]

5. What would need to happen to make a wider range of support available, including more time-intensive interventions? How could access to psychological therapies be improved?

6. What is the link between autism and depression? Misdiagnosis – are 'symptoms' of depression are actually 'traits' of autism (being quiet, withdrawn and needing to shut yourself away from the stimulus of people and the outside world) which would explain why trying to get someone out and mixing with people as a way out of depression would not work and in fact make things 100x worse?

7. How can others best support family members or friends with depression? What do people with depression find most helpful?

8. What are the specific problems that emerge from having a parent with depression, and what can be done to help counter these effects?

9. Can parents learn and teach healthy emotional behaviours and positive strategies (e.g. through therapy), even if they can't always do them themselves?

10. Can we ask GPs what training they received in mental health, whether they think it was adequate to prepare them for GP consultations, what more they would like to learn and what services do they wish they could refer patients to? Doing 6 months in inpatient psychiatry as an optional part of a rotation doesn't really prepare you for dealing with the majority of mental health issues in the community.
