# Supplementary material for: Depression detectives: piloting a methodology for online co-produced research
Source: Res Involv Engagem. 2026 May 18;12:62. doi: 10.1186/s40900-026-00889-2 (PMC13182061; doi:10.1186/s40900-026-00889-2)
Supplement: Supplementary file 3 — Supplementary Material 3: Co-produced Survey Questions and results. Includes Patient information sheet, survey questions asked and results as graphs & copies of the open text answers [file 40900_2026_889_MOESM3_ESM.pdf]

Additional Information 3

Co-produced Survey Questions and results.
Includes Patient information sheet, survey questions asked and results as graphs & copies of the open text answers.

Participant Information Sheet

Before you joined Depression Detectives, you read an information sheet
(You can see a copy of that at: https://edinburgh.onlinesurveys.ac.uk/depression-detectives)

Then you were agreeing to help **design** some research. Now you are about to **take part in** that research, which is a slightly different thing. So this new information sheet is just to make that clear, and check you are OK with it.

Research question: Are episodes of depression always visible in medical records and if they aren't, what are the reasons?

Methods:

1. Data Science – researchers are analysing UK Biobank data on our behalf.
2. Focus group discussions & polls in our private Facebook group
3. **This anonymous survey**

By completing this survey, you are agreeing to become a Research Participant.
Your responses to the questions that we ask here, will form part of our research results.

Anonymity
We will not ask you to provide any identifying personal details.
We also ask that you do not write anything that could identify you or others (e.g. your address or name of your doctor) into the text boxes.

If there is a question that you do not wish to answer, just miss it out.

What will happen to the survey results (data).

The results of this survey will be analysed by the Depression Detectives group and by University of Edinburgh researchers and may be written up externally as blog posts, reports, journal papers etc. Full answers will only be seen by staff, and will be checked and randomised before anything is shared in the group.

Because this is research data, it will be kept indefinitely.
However, if you wish to withdraw your answers, please let Iona know asap (iona.beange@ed.ac.uk) and she will do her best to delete your data (as the survey is anonymous, we will only be able to do this if you can tell us what you wrote in the free text boxes or what day/time you completed it).

Data Protection

Your data will be processed in accordance with Data Protection Law.

WHO CAN I CONTACT?

If you have any further questions about the study, please contact the project lead, Iona Beange, Knowledge Exchange and Impact Officer, University of Edinburgh [redacted]

If you wish to make a complaint about the study, please contact: Public Engagement with Research Manager, University of Edinburgh. Email: [redacted]

For general information about how we use your data go to: https://www.ed.ac.uk/records-management/privacy-notice-research

The Samaritans (www.samaritans.org) are a national listening service. If you feel triggered by this form, please consider contacting them for support. Phone 116 123

I have read and understood the information sheet and consent to take part in the project

Yes / No

I confirm that I am an existing member of the Depression Detectives Facebook Group

Yes / No

(No to either of these re-routes out of the survey).

Depression Detectives Research Survey

General information

26 responses (N = 26)

Do you experience episodic or chronic depression?

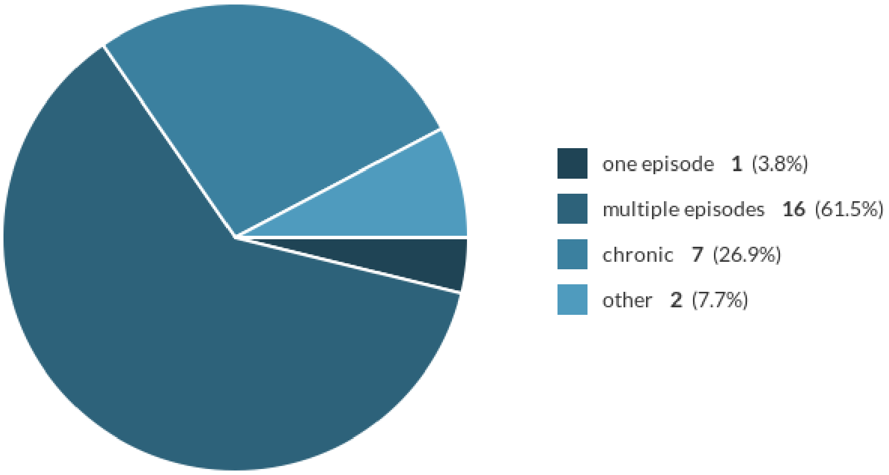

Other:

- I experienced depression from childhood until a couple of years ago and now (age 44) consider myself cured. The pattern was that I always had background levels of depression but experienced episodic relapses, but until 2 years ago I would say I was chronically depressed for as long as I can remember.

- These "episodes" I have don't have clear beginnings or ends

Do you feel like you fully recovered in between episodes?

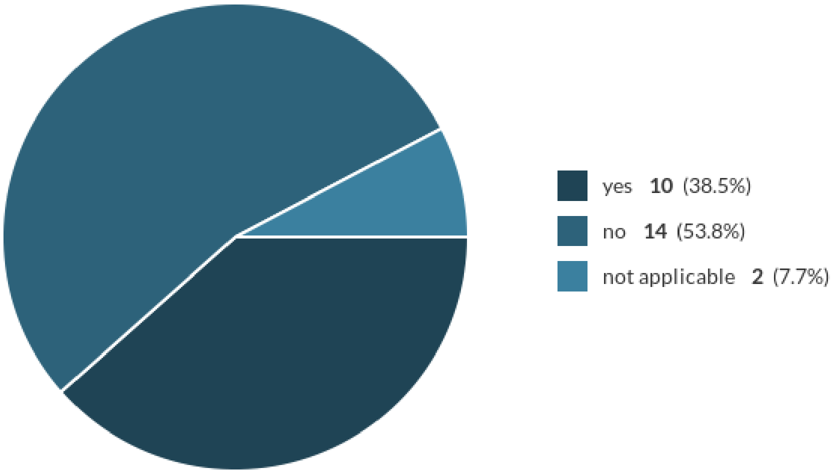

Can you remember how many episodes of depression you have had?

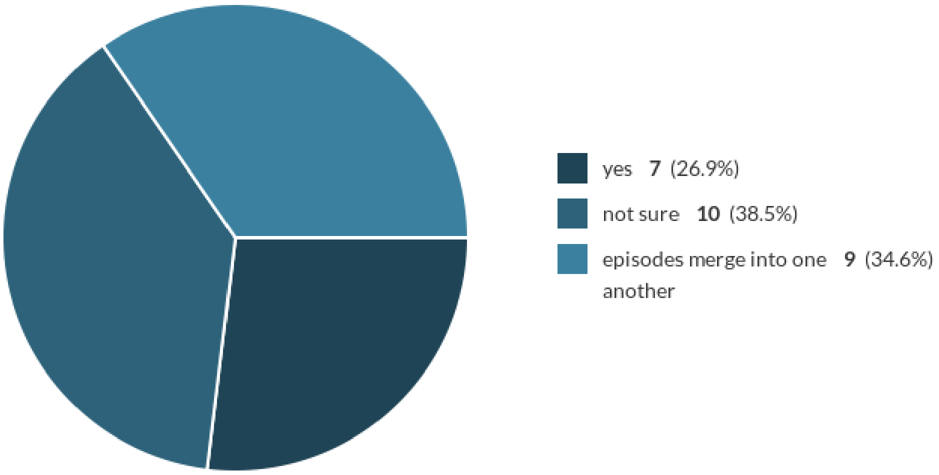

Enter the approximate number of episodes you can remember

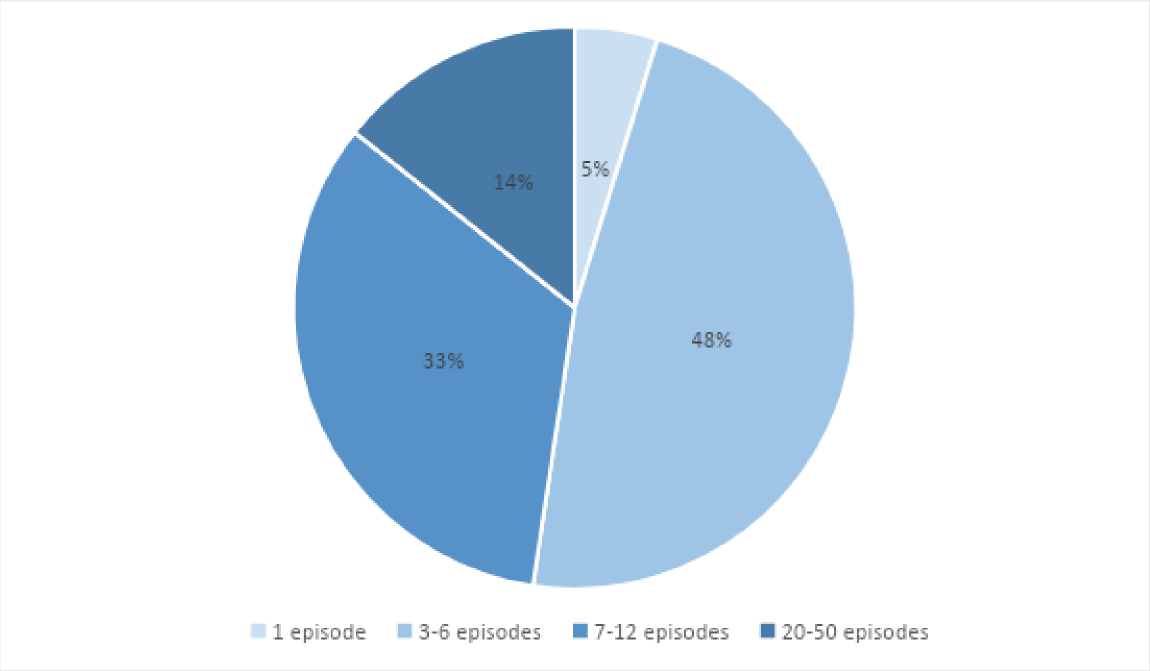

NUMBER OF EPISODES	NUMBER OF PARTICIPANTS
**1**	1
**3**	3
**4**	1
**5**	4
**6**	2
**7**	1
**8**	1
**10**	1
**12**	4
**20**	1
**30**	1
**50**	1

Have you been to the doctor’s with any of the episodes you’ve experienced?

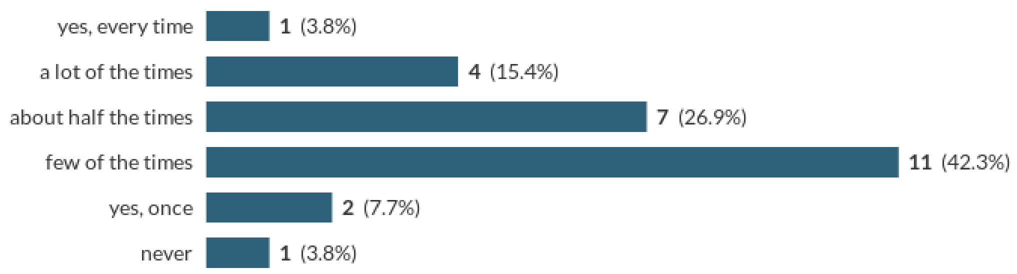

If you have been to the doctor’s with some of the episodes you’ve experienced, what was the pattern?

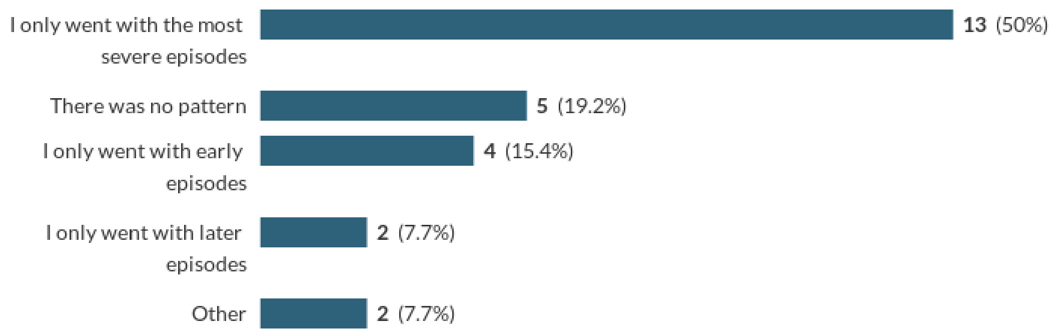

Other:

- I don't have episodes. It is a chronic condition. This entire survey is based on the assumption that depression occurs in "episodes", which may be the case for some people but excludes those for whom it is a chronic condition.
- When I knew how to access mental health support elsewhere (e.g.. privately or through work) then I didn't go to the GP. Only went to GP when I didn't know what else to do.

7. Thinking back to your FIRST experience of depression, who managed that episode/ helped you?

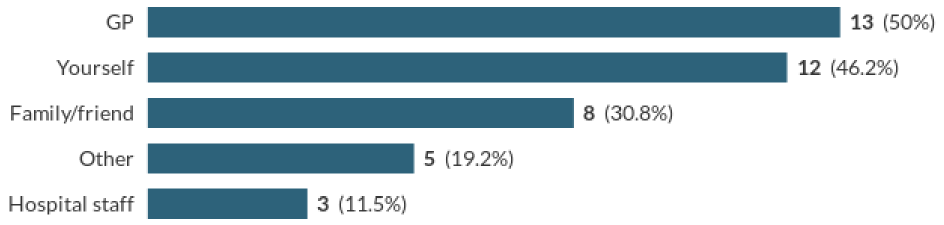

Other:

- A member of support staff at uni
- Counselling referred by GP
- University counselling service
- Was not diagnosed until ten years later

If you have contacted your GP about depression, have your experiences of going to the GP been overall positive, mixed, or neither.

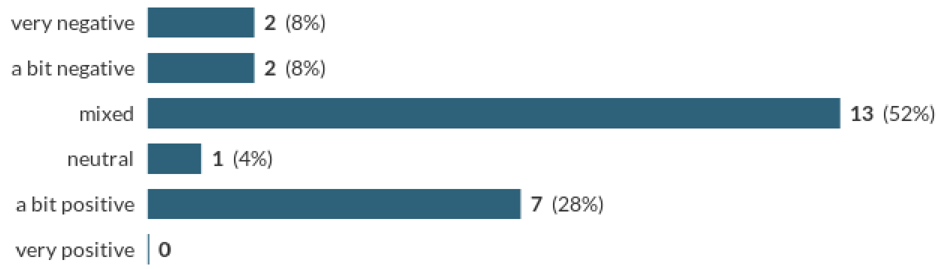

When you first sought your GP's help with depression, did they ask about previous episodes and past mental health?

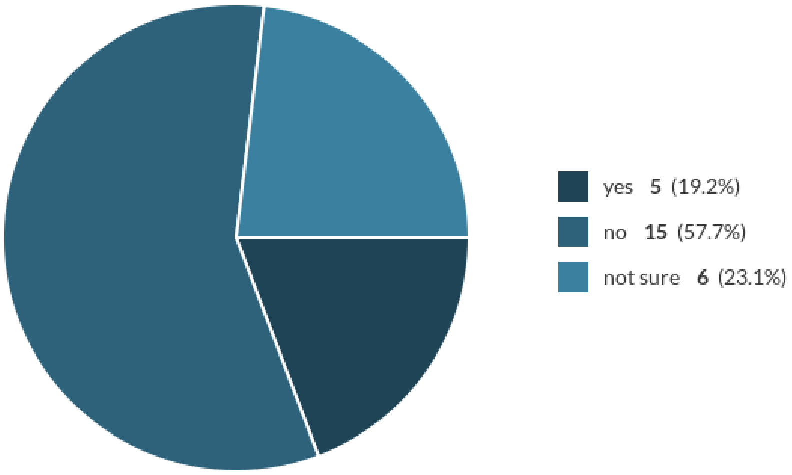

Have you ever had episodes of depression that you didn't go to the doctor with?

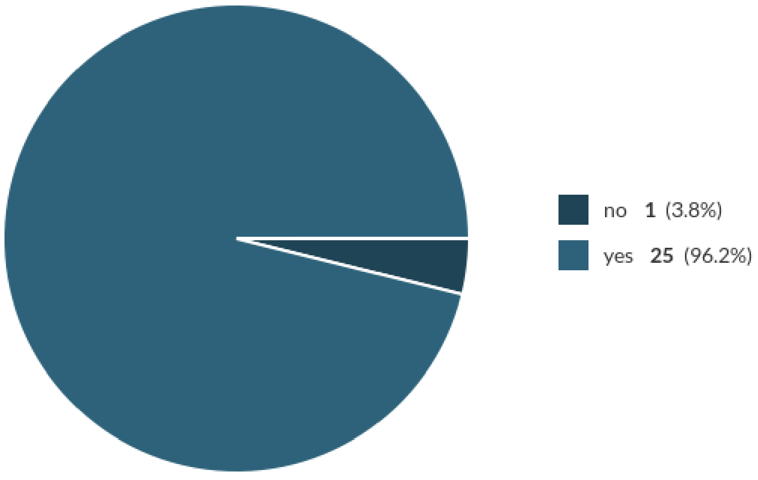

If there were episodes you didn’t go to the doctor with, why was that?

Reason for not going to the GP	Number of Participants
Didn’t think I was depressed ‘enough’ to justify going to doctor	18
I just thought what I felt was part of who I am, rather than it being an illness	15
Hoped it would pass by itself	12
I did not feel worth bothering the GP (initially or repeatedly); I feared being classed as a time waster or to be seen as a burden	12
I did not believe that the GP would be helpful, because the GP would not be able to give me enough time to explore what is going on and what I need	11
I did not believe that the GP would be helpful, because I thought other options not on offer from GP would be more useful	11
I was ashamed about not being able to cope	11
I am worried about being prescribed medication, as I don’t think they will treat the root of the problems	11
I did not have the energy to seek help	10
I felt very uncomfortable talking about my feelings or struggles	9
I did not believe that the GP would be helpful, because I had negative past experience with GPs who was unhelpful	9
I didn’t feel any hope that anything could make it better, at the time	9
I am worried about being prescribed medication, as I worried about side-effects	8
I was worried about work implications	8
I didn’t want ‘mental illness’ on my medical records	8

I felt it was related to a situation in my life at the time (e.g., grief) and would pass	7
I did not know that I was depressed, that what I was feeling is depression, and that a GP could help with what I am experiencing	7
I did not want to admit something was wrong or an episode is starting	7
I didn’t feel worthy of medical treatment and getting better	7
I was worried about pressures /cultural/ family perceptions/ stigma	7
I did not believe that the GP would be helpful, because I thought they will only offer drugs and nothing else	6
I did not believe that the GP would be helpful, because I felt too vulnerable	6
I didn’t want to make depression more real by giving it a name or saying it aloud	6
I am worried about being prescribed medication, as I don’t like taking medication	6
I am worried about being prescribed medication, as I worried about having to take it forever or being dependent	6
I was worried about being seen as someone to be pitied	5
I felt having mental health issues was shameful	5
I am worried about being prescribed medication, as I previously tried them and found them ineffective	5
I would have needed someone else to tell me to go or to make an appointment for me	4
I did not believe that the GP would be helpful, because consulting with a GP had caused me more pain before	4
I did not believe that the GP would be helpful, because I did not have a regular GP that I trust	4
I felt it was a natural process and wanted to allow my depression to run its course	4

Other reasons*	3
it was difficult to make an appointment as talking to the receptionist was hard	2
I did not believe that the GP would be helpful, because of what I heard from others or thought would happen	2
I did not believe that the GP would be helpful, because I could not choose which doctor to see, and didn't want to speak with the one that was given to me	2
I did not believe that the GP would be helpful, because the doctor seemed so different to me, I wasn't sure if we could relate to each other and the doctor would understand	1
Someone prevented me from going to the doctor about it	0

*Other reasons:

- Didn't realise it was depression
- I was looking after my mentally ill son and didn't want to focus on myself. Then didn't go to GP due to Covid pandemic.
- I thought it was my fault and I should not need a doctor

Which of the reasons in the previous question was the most important for you?

Because the GP only ever offered drugs and that didn't solve my problems but I knew I could seek other forms of therapy which were more likely to help

---

Feeling that the drugs were not really going to make me better or sort the underlying issues

---

Because I thought other options not on offer from GP would be more useful

---

Probably not having a relationship with trusted GP. I hardly went to doctors about anything and while I was registered with GP at unil I think I only made an appointment twice for things I knew I absolutely needed from them.

Felt uncomfortable

I didn't know it was depression as I don't feel worthy enough to get the attention

---

I just thought what I felt was part of who I am, rather than it being an illness

---

When I’m badly depressed I feel there’s no point in seeking help because it keeps happening. Even if I get medication and feel better, it’s only a matter of time before it happens again

---

Didn’t recognise that it was a medically treatable situation/bad enough I was in

---

I managed it myself

If you did not go to a GP, did you ever regret this afterwards?

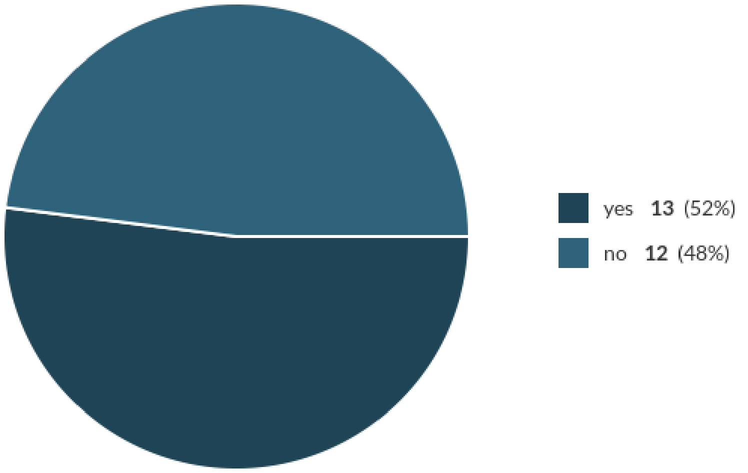

If you chose not to go to the doctor's, what did you do instead?

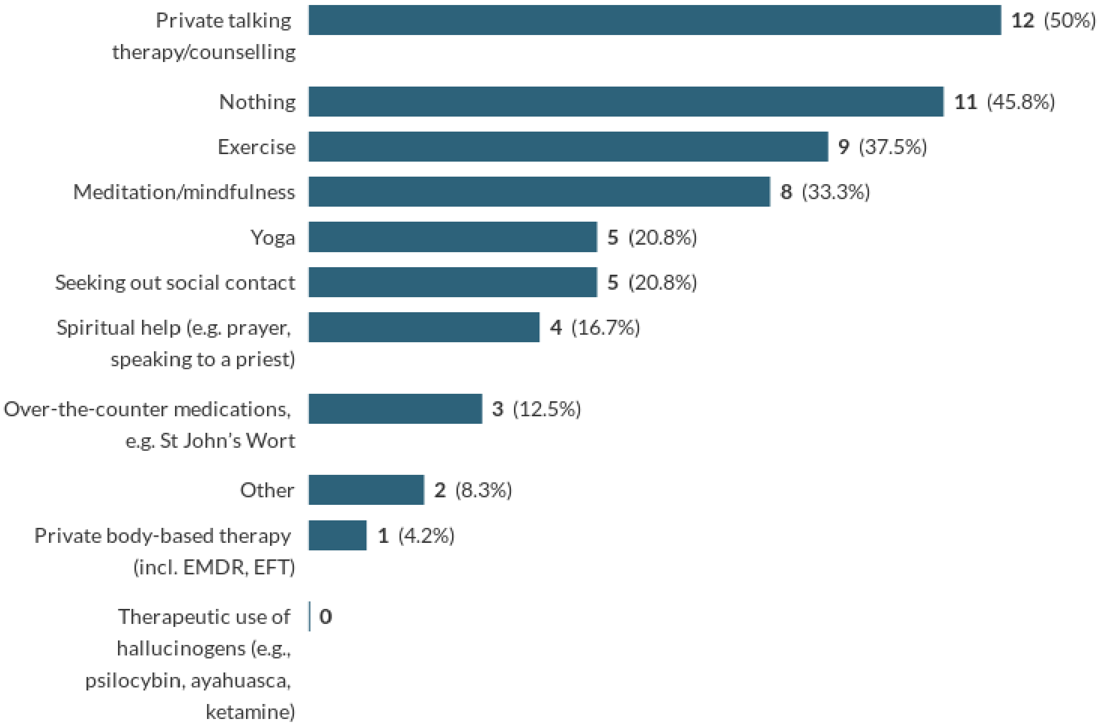

Other:

- learning positive psychology
- self-help books

Did you prefer the alternative options in the previous question, compared to going to the doctor?

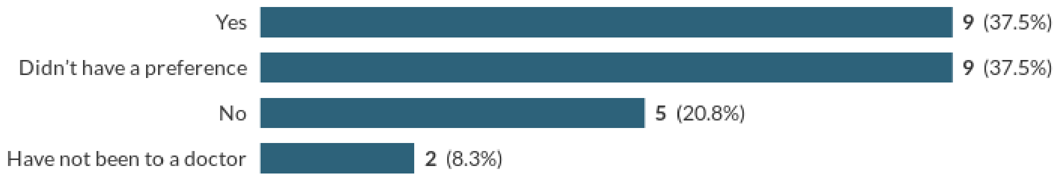

If so, what did you prefer about them

The doctor only gave pills, which may take away a bit of the pain but don't solve the underlying problem so it's always going to come back. Learning positive psychology as a means of addressing childhood trauma has enabled me to cure myself.

Much more time spent identifying the root of the problem, rather than just applying an emotional sticking plaster.

They seemed to address the root of the problem rather than just symptoms.
They helped me to grow rather than just cope.

I felt that talking therapy helped me to get to the bottom of why I felt the way I did. My therapist was also very good at helping me to understand my depression and understanding that it is an illness and not just who I am.

More holistic

Better quality of therapy and quicker access (immediate).

Easy to do myself in my own time on my own terms

Could have not ended up in such a bad way

Having the time to actually talk about things and explore things

What would you do for future episodes?

Go to GP if not during a pandemic!

Go to GP. I tested in use of psychedelics.

Doctor

I might go to the GP. I like my current GPs (even though I don't go that regularly). I think biggest barriers right now are busyness and being reluctant to admit I have a problem.

Mixture of doctor to get medication and counselling and life style changes to try to prevent another episode in the first place

---

See help earlier

I would try to catch them early before I spiral down.
If I got depressed, I would probably go for private counselling

---

Private or through employment.

I would seek to manage it myself and go to the GP if I felt that I needed time off work or that medication might help.

---

deal with this on my own or kill myself

Yoga, relaxation, private counselling

I don’t know

Depends on severity and cause plus engineering time. My trusted GP only works 2 fixed days per week, I have to get a day off, phone on the day and hope there’s an appointment free, navigating this when depressed is very challenging.

I will only seek help from a Dr if it becomes too difficult to manage or I need time off work

Remember that I had been there before and remind myself I improved by using some of the tools I had learned

Doctor, but psychiatry team unless GP was one I knew would be ok, as I have had bad experiences of being dismissed and told it wasnt that bad and I just needed to buck up because I could still read out a sentence when asked to despite not being able to concentrate enough to do simple calculations needed for work and being afraid I might kill myself so avoiding certain places I might be tempted.

Is there anything else you wanted to say to us?

I have been depressed in the past and became depressed after I retired in 2019. Also my adult son took an overdose twice and I was desperately worried about him. I had no support from the NHS due to confidentiality as he is an adult. I didn't know how to support him. I felt a fool for not realising what I was getting from work as I "hated" it. I didn't want my son to know I was depressed so didn't go to GP. Then didn't go due to pandemic. I think I should have gone. I don't want drugs but I know and trust some of the GPs at the practice so I am sure now taking to them would have helped.

I never used to believe that a cure was possible, thinking that this was just the way my brain worked due to my past experiences and there was nothing I could do about it. As a result of needing to find a better way of being so I could parent my children in a more positive way and not pass on the trans-generational trauma, I have managed to fight my way out of the cultural expectation that depression is a chronic illness and I have re-wired my brain. Through positive parenting, I discovered positive psychology techniques which have enabled me to cure my depression. I am not sure that this would ever have been available from the GP!

I've never formally been diagnosed with depression so perhaps none of my 'episodes' would quite fall into that category. I've had 'tough times' where I felt I needed professional help to cope and found this more effectively through work based counselling schemes.

This survey is not designed to be inclusive and simply assumes that everyone experiences depression as episodes. I think this is a bit problematic because that assumption means that depression is once again not being considered as a chronic condition which requires ongoing treatment/medication but instead simply comes and goes, and if that is how doctors assume depression occurs it is hardly surprising that people are often dismissed or not treated appropriately with a longterm view, because the thinking is "well you're going through a bit of a rough patch but it will get better in a few weeks/months, all we need to do is get you through this spell". My depression is not something that goes away if you just wait it out or treat it as you would an infection or a sprained ankle.
