# Supplementary material for: Depression detectives: piloting a methodology for online co-produced research
Source: Res Involv Engagem. 2026 May 18;12:62. doi: 10.1186/s40900-026-00889-2 (PMC13182061; doi:10.1186/s40900-026-00889-2)
Supplement: Supplementary file 4 — Supplementary Material 4: Evaluation Form Questions Includes the questions presented to participants and researchers about their experiences of the project [file 40900_2026_889_MOESM4_ESM.pdf]

Additional Information 4

Evaluation Form Questions

Includes the questions presented to participants and researchers about their experiences of the project.

Participants Evaluation Questions

Do you work in a mental health-related role or are you studying a mental health-related subject? (e.g. a researcher, counsellor, mental health nurse, work for a mental health charity or have some other mental health related role)

- Yes
- No

Which of these statements best describes your attitude towards mental health research / the science around depression?

- I am against / distrust it /feel negatively towards it.
- I don't think about it at all
- I have some awareness of it, but I wouldn't look it up.
- I sometimes come across things
- informed by it, but I wouldn't look for them.
- I would like to know more about it, but the information is hard to find or understand.
- I sometimes look it up / read or watch things informed by it.
- I regularly look it up / read or watch things informed by it.
- I am very up-to-date with it and often read about it /articles informed by it.

Do you feel that research / scientific information is relevant to your own personal management of your depression?

- Definitely not
- No
- I'm not sure
- Perhaps
- Yes, definitely

How comfortable do you feel reading research papers or articles/books that describe scientific Research. I feel:

- Very comfortable
- comfortable
- somewhere in between
- unsure
- intimidated

If you were not sure about something a healthcare provider says or have a different opinion, would you be able to ask or challenge them?
Asking of challenging a healthcare provider feels...

- very comfortable
- comfortable
- somewhere in between
- reluctant
- would stay silent

Further thoughts on this question?
Free text box

Why did you get involved with this project?
Free text box

Did participating in this project meet your expectations? If so, why? if not, why not?
Free text box

How useful did you find the following activities?

Polls

- Extremely useful
- Useful
- Neither useful or not
- Not very useful
- Not at all useful
- I didn’t do this

Taking part in discussions in the group

- Extremely useful
- Useful
- Neither useful or not
- Not very useful
- Not at all useful
- I didn’t do this

Taking part in Q&A with researchers

- Extremely useful
- Useful
- Neither useful or not
- Not very useful
- Not at all useful
- I didn’t do this

Reading transcripts of Q&A with researchers

- Extremely useful
- Useful
- Neither useful or not
- Not very useful
- Not at all useful
- I didn’t do this

Designing an experiment

- Extremely useful
- Useful
- Neither useful or not
- Not very useful
- Not at all useful
- I didn’t do this

Taking part in focus group discussions

- Extremely useful
- Useful
- Neither useful or not
- Not very useful
- Not at all useful
- I didn’t do this

Filling in research survey

- Extremely useful
- Useful
- Neither useful or not
- Not very useful
- Not at all useful
- I didn’t do this

Finding out about the results of the research we did together

- Extremely useful
- Useful
- Neither useful or not
- Not very useful
- Not at all useful
- I didn’t do this

Have you worked with a researcher before this project?

- Yes
- No

Having participated in this project, how would you describe the research process in your own words?
Free text box

In terms of your personal experience, how important is it for you to be able to ask researchers questions about depression? Why?
Free text box

What impact did participating in this project have on you and your management of your own depression?
Free text box

Has participating in the project prompted you to explore the subject further on your own? If so, how?
Free text box

Is there anything else you'd like to tell us about your experience of the project or anything you think we should consider for future sessions?
Free text box

What is your full name? (We will anonymise this survey, but it will help us to have your name in order to compare what you said in the sign up survey with what you said here. However, this question is optional and you can choose not to say your name.)

What is your gender?

- male
- female
- other
- prefer not to say

What is your highest science qualification?

- grade/GCSE
- Higher/A level
- Degree/HND/Similar
- Postgraduate qualification

Can you give us the first four digits of your postcode?
Text box

Researchers Evaluation Questions

Taking part in Depression Detectives felt…(tick as many as you like)

- Good
- New
- Fun
- Hard
- Thought-provoking
- Frantic
- Stressful
- Unsettling
- Productive
- Inspiring
- Easy
- Familiar
- Exhilarating
- Other

If you selected other, please specify
Free text box

How confident do you feel about engaging the public after this experience?

- More confident
- Less confident
- About the same

In your own words, what was your role in the project?
Free text box

In your own words, what do you think this public engagement project was trying to do?
Free text box

What was the worst thing about taking part?
Free text box

What was the best thing about taking part?
Free text box

Did you learn anything that surprised you?
Free text box

Did you learn anything that will influence your research?
Free text box
